# Advances in ex vivo expansion of hematopoietic stem and progenitor cells for clinical applications

**DOI:** 10.3389/fbioe.2024.1380950

**Published:** 2024-05-23

**Authors:** André Branco, Janakiram Rayabaram, Cláudia C. Miranda, Ana Fernandes-Platzgummer, Tiago G. Fernandes, Suchitra Sajja, Cláudia L. da Silva, Mohan C. Vemuri

**Affiliations:** ^1^ Department of Bioengineering and Institute for Bioengineering and Biosciences (iBB), Instituto Superior Técnico, Universidade de Lisboa, Lisbon, Portugal; ^2^ Associate Laboratory i4HB—Institute for Health and Bioeconomy, Instituto Superior Técnico, Universidade de Lisboa, Lisbon, Portugal; ^3^ Protein and Cell Analysis, Biosciences Division, Invitrogen Bioservices, Thermo Fisher Scientific, Bangalore, India; ^4^ AccelBio, Collaborative Laboratory to Foster Translation and Drug Discovery, Cantanhede, Portugal; ^5^ Thermo Fisher Scientific, Frederick, MD, United States

**Keywords:** hematopoietic stem and progenitor cells, *ex vivo* expansion, clinical translation, signaling, metabolism, potency

## Abstract

As caretakers of the hematopoietic system, hematopoietic stem cells assure a lifelong supply of differentiated populations that are responsible for critical bodily functions, including oxygen transport, immunological protection and coagulation. Due to the far-reaching influence of the hematopoietic system, hematological disorders typically have a significant impact on the lives of individuals, even becoming fatal. Hematopoietic cell transplantation was the first effective therapeutic avenue to treat such hematological diseases. Since then, key use and manipulation of hematopoietic stem cells for treatments has been aspired to fully take advantage of such an important cell population. Limited knowledge on hematopoietic stem cell behavior has motivated in-depth research into their biology. Efforts were able to uncover their native environment and characteristics during development and adult stages. Several signaling pathways at a cellular level have been mapped, providing insight into their machinery. Important dynamics of hematopoietic stem cell maintenance were begun to be understood with improved comprehension of their metabolism and progressive aging. These advances have provided a solid platform for the development of innovative strategies for the manipulation of hematopoietic stem cells. Specifically, expansion of the hematopoietic stem cell pool has triggered immense interest, gaining momentum. A wide range of approaches have sprouted, leading to a variety of expansion systems, from simpler small molecule-based strategies to complex biomimetic scaffolds. The recent approval of Omisirge, the first expanded hematopoietic stem and progenitor cell product, whose expansion platform is one of the earliest, is predictive of further successes that might arise soon. In order to guarantee the quality of these *ex vivo* manipulated cells, robust assays that measure cell function or potency need to be developed. Whether targeting hematopoietic engraftment, immunological differentiation potential or malignancy clearance, hematopoietic stem cells and their derivatives need efficient scaling of their therapeutic potency. In this review, we comprehensively view hematopoietic stem cells as therapeutic assets, going from fundamental to translational.

## 1 Introduction

Hematopoietic cell transplantation (HCT) is the most promising therapy for treating many hematological diseases. Since its establishment in 1957 by E. Donnall Thomas (Thomas et al., 1957), infusion of healthy marrow cells into radiation-treated diseased bone marrow (BM) has been able to cure up to 1.5 million patients, according to the Worldwide Network of Blood and Marrow Transplantation (WBMT) (Niederwieser et al., 2021). Each year, an average of 84,000 transplants are successfully performed (Niederwieser et al., 2021). Being the first clinically employed stem cell-based therapy (close to reaching its 70th anniversary), HCT has had to deal with treatment obstacles from facing donor cell rejection, leading to graft failure, to having donor immune cells attacking the tissues of the receiving patient, triggering graft vs host disease (GVHD). Consequently, HCT has had to outmaneuver and innovate over the years. With the discovery of the human leukocyte antigen (HLA), immunological identity was unlocked, and mechanisms of cell and tissue compatibility were finally begun to be understood (Petersdorf, 2004). Rejection and GVHD could now be identified and comprehended as biological responses. HLA-matching between patients and potential donors is indispensable in an HCT scenario and is one of the main criteria for determining donor eligibility (Gupta and Wagner, 2020). Since HCT was historically allogeneic in its infancy, HLA-matching highlighted the advantage of having a full HLA-match in autologous HCT. Nevertheless, in hemato-oncology, alloreactivity in allogeneic HCT is also responsible for a beneficial graft vs leukemia (GVL) effect. Being an essential ally in fighting cancer, GVL emerges when donor lymphocytes can recognize malignant recipient cells, further contributing to disease remission (MATHÉ et al., 1965; Dickinson et al., 2017). Interestingly, allogeneic and autologous HCT have gained value and indications for specific diseases. As of 2019, 53.5% of all HCT were allogeneic, but both have increased between 2006 and 2016 (allogeneic—89.0%; autologous—68.9%) (Niederwieser et al., 2021). While leukemias and non-malignant hematological disorders (e.g., Hemoglobinopathies or autoimmune disorders) are preferentially treated with allogeneic HCT, patients with lymphoproliferative disorders and solid tumors are mostly subjected to autologous HCT (Niederwieser et al., 2021).

**FIGURE 1:**
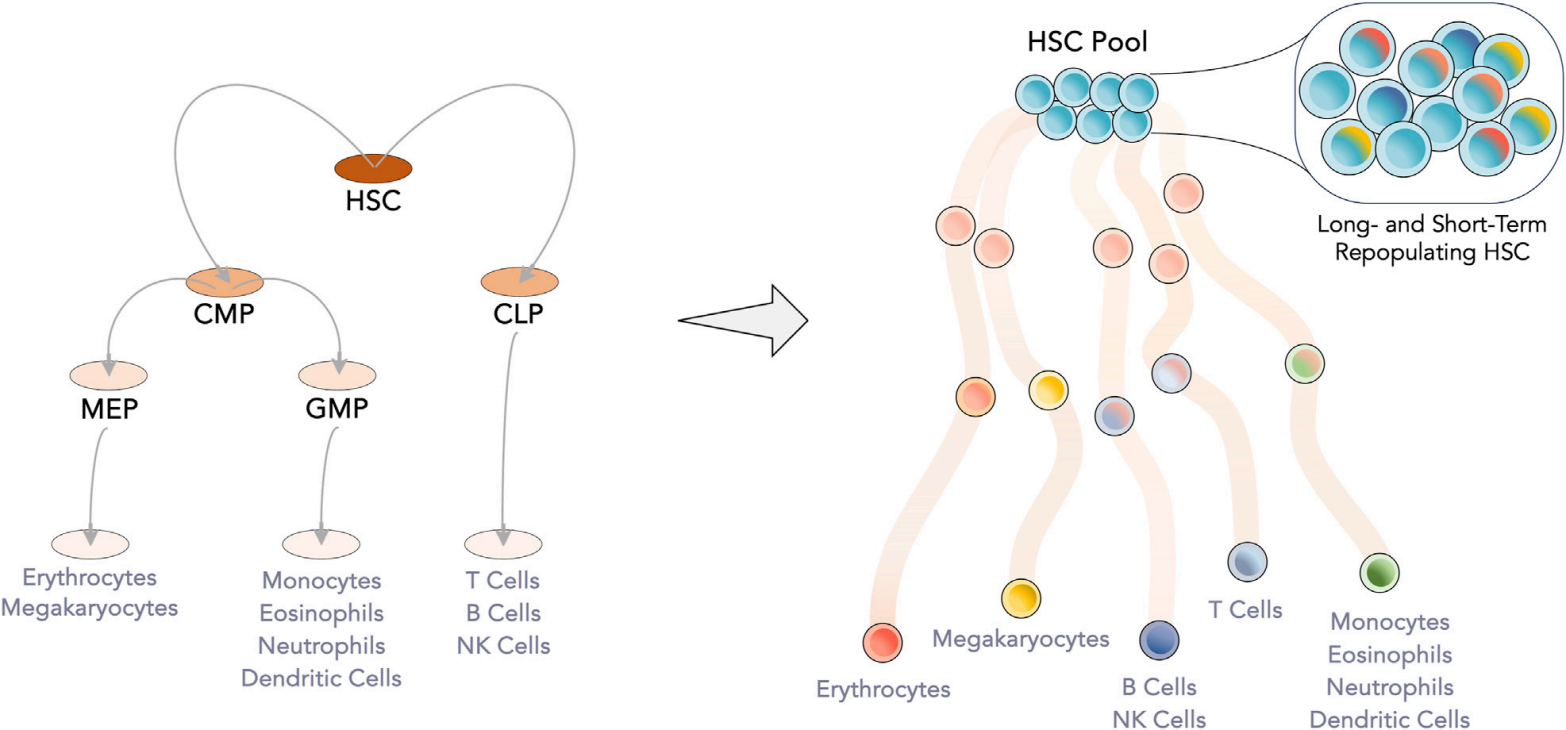
Evolution of the hematopoietic tree. Gained knowledge of the hematopoietic system has led to the readjustment of the hematopoietic tree, from a set of discrete populations and clear separation between the myeloid and lymphoid lineages to a more continuous model. At the apex, the limited HSC pool, which was considered to be homogeneous, is now known be transcriptomically heterogeneous with individual cells having a bias towards a specific lineage. Intermediate progenitor populations have begun to be considered as transient junctions along the differentiation pathway. CLP—Common lymphoid progenitor; CMP—Common myeloid progenitor; GMP—Granulocyte-monocyte progenitor; HSC—Hematopoietic stem cell; MEP—Megakaryocyte-erythrocyte progenitor.

Hematopoietic stem cells (HSC) and hematopoietic progenitor cells (HPC) are the leading cell players of HCT. At the apex of the hematopoietic system, HSC are responsible for the lifelong supply of blood cells. Between their self-renewal and differentiation, the most primitive cells must secure steady production of two main branches, lymphoid (making up the adaptive immune system, with T- and B- cells) and myeloid (encompassing oxygen transport with erythrocytes, coagulation with platelets and the innate immune system with monocytes and granulocytes). Initially conceived as a rigorous hierarchy with discrete differentiation stages, the hematopoietic tree has been suggested to be much more fluid with continuous differentiation avenues (Laurenti and Göttgens, 2018) (Figure 1). Either by tracking the ratio of active X-chromosomes of paternal and maternal origin in women or by inferring HSC clonal dynamics from somatic mutations, a limited hematopoietic stem pool between 3,000 and 200,000 HSC has been proposed to maintain human steady-state hematopoiesis (Catlin et al., 2011; Lee-Six et al., 2018).

Although infusion of HSC and subsequent engraftment are the critical therapeutic objective in HCT, side objectives, from avoiding thrombocytopenia to accomplishing timely immune reconstitution, are essential for early patient survival. After myeloablation, the coordinated production pipeline of the hematopoietic system is disrupted. Engrafted HSC can reinstate steady-state hematopoiesis, while more committed HPC ensure short-term hematopoietic reconstitution. Therefore, HPC also serve an essential purpose, and their role in the initial success of an HCT might be even more significant than their more undifferentiated precursor (i.e., HSC) (Biasco et al., 2016). The ideal hematopoietic graft should include a mixture of hematopoietic stem and progenitor cells to reduce transplant-related mortality, safeguarding both long-term and short-term hematopoiesis. Of notice, fully differentiated cells present in the graft, especially of the immune system, can increase the probability of GVHD and other immune-mediated complications (Z. Li et al., 2016).

The success of HCT is multi-factorial, with multiple approaches being explored to improve such an impacting therapy. Concerning donor-recipient compatibility, high-resolution HLA-typing has allowed even more significant and excellent immunological compatibility. Permissive mismatches in HLA-DPB1 have been shown to cause limited alloreactivity (promoting GVL while shielding from GVHD), which has led to their inclusion in the donor selection criteria (Fleischhauer et al., 2012; Meurer et al., 2021). Also, the clinical impact of minor histocompatibility antigen mismatching is poorly understood, being a target for research to improve the outcome of allogeneic transplants (Martin et al., 2017; Nie et al., 2021). Concerning pre-HCT patient conditioning, for certain diseases, the development of non-myeloablative and reduced-intensity regimens has significantly reduced toxicity and increased the range of eligible ages for HCT (Gyurkocza and Sandmaier, 2014; Jethava et al., 2017). Patients aged ≥70 years who did not have access to this treatment option beforehand are now undergoing allogeneic or autologous HCT with steady growth over the years (D’Souza et al., 2020). At the cellular level, improving stem/progenitor cell homing and engraftment has been similarly explored as a strategy. Upon the identification of the CXCR4/CXCL12 axis as a primary mechanism for cell homing, the use of glucocorticoids, stabilization of HIF-1α, or incorporation of CXCR4 into lipid rafts have contributed to promote this chemotaxis/migration axis (Huang and Broxmeyer, 2019).

While each of the abovementioned approaches has relevance, graft cell dose (i.e., per Kg of body weight) is a dominant factor affecting HCT outcome. Patients require a specific number of infused cells to ensure successful engraftment. Reaching clinically relevant cell doses is more complicated depending on the hematopoietic cell source, limiting HCT outcomes. Poorly mobilized peripheral blood, or umbilical cord blood (UCB) suffers from this drawback, requiring, respectively, mobilization improvement strategies or a double unit cord blood transplant (DUCBT) if wanting to treat pediatric patients of higher weight or adults (Brunstein et al., 2010; To, Levesque, and Herbert, 2011). Development of *ex vivo* expansion platforms for HSC and HPC has unsealed immense potential by directly tackling cases of low cell dose, making it possible to increase hematopoietic cell number *in vitro* before administration into patients (Wilkinson, Igarashi, and Nakauchi, 2020). This disruptive technology will have a broader impact on HCT since increasing the number of primitive cells would drastically increase access to UCB units, with less stringent HLA-matching requirements, improve overall HCT outcomes with higher doses of hematopoietic stem and progenitor subpopulations, and even accelerate immune reconstitution by facilitating the adoption of donor lymphocyte infusions (DLI). Gene therapies based on HCT will also benefit from strategies able to overcome the obstacle of HSC and HPC quantity, since low efficiencies of genetic modification platforms and cell losses during transduction/transfection protocols would usually challenge therapy viability (Zonari et al., 2017).

In this review, we will focus on developments and breakthroughs of *ex vivo* expansion of HSC and HPC for clinical application, also highlighting the latest discoveries in HSC biology (including metabolism), the importance of having a spectrum of hematopoietic cell sources, and a compilation of signaling pathways involved in HSC development and expansion.

## 2 Sources of HSC and HPC for clinical use

HSC and HPC can be harvested from different locations for later application in HCT and other curative therapies. Rigorous identification and characterization of isolated HSC and their progenitors is of major importance for research and clinical applications. The possibility of isolating and identifying cells with a suitable surface marker expression profile allows the study of HSC hierarchies, exact mechanisms involved in hematopoietic development, homeostasis, and differentiation, as well as their clinical use. There are approximately 20 different surface markers that can be exploited for the isolation and identification of human HSC such as CD33, CD34, CD49f, CD90, CD93, CD117, CD133, CD143, CD370, EPCR, and GPRC5C. The expression patterns (in percentage) of these surface markers vary based on the source of HSC. However, despite their contribution, there is always some degree of ambiguity in the field regarding the most relevant surface marker combination to identify HSC and HPC in different conditions. The significance and challenges with surface markers in the HSC hierarchy has been comprehensively described in a recent review (Anjos-Afonso and Bonnet, 2023).

As the homeostatic home of adult HSC, BM was the sole source of HSC and HPC for many years. Residing inside solid bone tissue, HSC and HPC populations dwell in an intricate web of spongy bone that compartmentalizes in different micro niches (Birbrair and Frenette, 2016). While these primitive populations were also found in peripheral blood, their number is residual and insufficient for clinical application. However, with better knowledge of HSC and HPC regulation and biology, spatial restriction of these cells in the BM was seen to be tightly regulated. Granulocyte colony-stimulating factor (G-CSF) and, more recently, Plerixafor came as disruptors of HSC/HPC retention through suppression of CXCL12/CXCR4, c-kit/SCF, and VCAM-1/VLA-4 axes (Greenbaum and Link, 2011; Bilgin and de Greef, 2016). Consequentially, mobilization of HSC and HPC to the peripheral blood was made possible, opening another alternative for cell harvest (Sheridan et al., 1992), in a less invasive way. With time, gained knowledge of developmental hematopoiesis pointed out that HSC and HPC might exist in other tissues, leading to the finding of these primitive cells in umbilical cord blood, as well as placenta, drawing enthusiasm towards exploiting a medical waste of easy procurement (Broxmeyer et al., 1989). Together, BM, mobilized peripheral blood (mPB), and UCB provide complementing options for HSC and HPC isolation, with differences impacting their appeal as a source for HCT. These hematopoietic cell sources, each with distinct properties and advantages, have made it possible for nearly any patient candidate for HCT to find a potential donor (Kindwall-Keller and Ballen, 2017).

Depending on donor availability and HLA-matching score, BM aspirates can be sourced for matched siblings, matched unrelated, and haploidentical transplants. These transplants have a moderate risk of developing GVHD since T cell maturation is done in the thymus instead of the BM. Many differentiated immune cells are in secondary hematopoietic organs or circulation (Velardi et al., 2021). Unsurprisingly, for matched unrelated and haploidentical transplants, BM-derived grafts resulted in a lesser incidence of chronic GVHD than mPB, with equivalent or improved survival measures (Anasetti et al., 2012; Srinivasan et al., 2022). BM grafts have also been reported to induce lower cytokine release syndrome, a life-threatening HCT complication that induces a systemic inflammatory response, compared to mPB grafts (4.9% vs. 19.5%, respectively) (Abboud et al., 2021).

When looking at mPB, the collection of HSC and HPC from this source has progressively gained popularity, being by far the preferred source in allogeneic HCT for adult patients (Auletta, Kou, and Shaw, 2021). Mobilization of HSC/HPC has practical benefits, with graft procurement becoming similar to a standard blood harvest for donation. BM stem/progenitor cells gradually migrate into circulation when mobilized, mixing with regular PB and their native hematopoietic populations. Thus, immunologically active cells are much more present in mPB grafts, increasing the threat of GVHD (Saraceni et al., 2015). This increased alloreactivity also has benefits, especially in allogeneic HCT for malignant diseases. GVL is enhanced in mPB grafts and helps to reduce relapse risk (Byrne et al., 2016). To push the balance between GVHD and GVL towards the latter, increased mobilization of regulatory T cells (Treg) might improve patient protection against GVHD while maintaining a valuable GVL effect (Pastore et al., 2012).

When sourcing HSC and HPC from UCB, grafts potentially benefit from inherent tissue primitiveness. As such, GVHD-contributing populations are rare, providing an important option that can partially circumvent such a transplant-related complication (Sanchez-Petitto et al., 2023). For instance, incidence of chronic GVHD was seen to be significantly lower when UCB cells (5.8%) are used compared to mPB (10.1% - mismatched unrelated donor and 11.5% matched unrelated donor) in the therapeutic context of acute myeloid leukemia (Malard et al., 2015). Its attractiveness is also due to its collection as a source, which involves recovering blood from a severed umbilical cord, existing no risk for the mother or child. Moreover, such a naïve graft also allows loosening HLA-matching requirements, with recent selection guidelines for HCT stating at least a 4/6 HLA-match or 4/8 for high-resolution HLA-match (Dehn et al., 2019). On the other hand, compared to the adult sources, patients submitted to HCT performed with UCB cells have the longest time of engraftment and hematopoietic reconstitution due to this same graft trait (Panch et al., 2017). Without more differentiated progenitors, transplanted HSC and HPC will take longer to replenish the entire hematopoietic system until steady-state hematopoiesis can be reached. Interestingly, UCB-derived CD34^+^ cells were compared with mPB concerning their reaction to a simulated combined inflammatory response, with UCB-derived cells exhibiting a higher survival (Forte et al., 2018). Limited collection volume (around 100 mL) also restrains the use of an increased CD34^+^ cell dose, having pressured the development of *ex vivo* expansion strategies to bypass this obstacle (Talib and Shepard, 2020). A recent systematic review has confirmed the clinical advantages of HCT done with expanded HSC and HPC from UCB, though alerting towards the need for long-term outcome data (Saiyin et al., 2023).

Efforts to expand available HSC and HPC have resulted in significant gains in generating functional stem/progenitor cells from human pluripotent stem cells (hPSC) (Demirci, Leonard, and Tisdale, 2020). Combining genome editing techniques with human-induced PSC (hiPSC)-generated HSC has shown promise, particularly for nonmalignant hematologic disorders. However, successful generation of engraftable HSC from hiPSC has been challenging, requiring forced exogenous expression of transcription factors and often resulting in teratoma formation upon *in vivo* administration in animal models (Demirci, Leonard, and Tisdale, 2020). Hematopoietic development from hiPSC was elucidated by demonstrating that primitive and definitive hematopoietic programs both arise through hemogenic endothelial intermediates and originate from KDR^+^CD34^−^CD144^−^ progenitors, with CD235a distinguishing them (Sturgeon et al., 2014). The role of stage-specific activin-nodal signaling and Wnt–β-catenin pathway inhibition was seen to be essential for primitive hematopoiesis (KDR^+^CD235a^+^), while Wnt–β-catenin signaling during the same period is crucial for definitive hematopoiesis (KDR^+^CD235a^−^), thus providing targeted differentiation strategies for generating distinct hematopoietic progenitors from hiPSC. A robust protocol for the generation of HSC from hiPSC through specific stages, including mesoderm induction, hematovascular development, and the emergence of hematopoietic progenitors, has been recently described (Nafria et al., 2020). Although using an inducible gene expression strategy (based on four transcription factors—*SCL*, *LMO2*, *GATA2*, *ETV2*), another group has also contributed with a robust HSC differentiation protocol (Lange et al., 2020). hiPSC-derived HSC mirrored main hematopoietic developmental stages during their generation and gave rise to differentiated hematopoietic cell populations. HSC from different developmental stages were also mapped and compared with hiPSC-derived HSC (Calvanese et al., 2022). Specifically, pre-hemogenic endothelium (HE) and HE *in vitro* displayed either undetectable or weak expression of ALDH1A1, Wnt, and TGFβ/BMP inhibitors, which were found to be crucial regulators of endothelial-to-hematopoietic transition (EHT) *in vivo*. These discrepancies in signaling pathways during EHT raised concerns about the complete maturation of HSC in the *in vitro* model. Identifying these molecular bottlenecks is necessary to enhance the efficiency of generating fully functional HSC for different biomedical applications including fundamental studies of developmental hematopoiesis, disease modeling, and drug discovery, as well as towards therapeutic application for malignant and nonmalignant hematological disorders (reviewed in (Demirci, Leonard, and Tisdale, 2020)).

## 3 Origins of HSC during development

Homeostasis of hematopoietic stem cells and their consequent differentiation into various blood cell lineages is intrinsically regulated. Several studies to date, employing different animal models, from invertebrates to vertebrates, had connected the dots in hematopoiesis, where some of the events, surface markers and mechanisms, are not fully recapitulated in humans. Pioneers in the field highlighted several aspects of HSC biology, ranging from ontogeny to aging and leukemia transformation, emphasizing the development and characterization of human HSC. 1 million mature blood cells have been shown to be produced per second (Ogawa, 1993). There are different types of blood cells: erythrocytes, megakaryocytes/platelets, monocytes, macrophages, granulocytes, mast cells, T and B lymphocytes, NK cells, and dendritic cells. The source of these cells was researched and Till and McCulloch proposed the ‘stem cell’ after their studies on the regeneration of the blood system *in vivo* (Till and McCulloch, 1961). Two types of HSC, long-term HSC, (LT-HSC) and short-term HSC (ST-HSC), have been demonstrated (Spangrude, Heimfeld, and Weissman, 1988). The LT-HSC are on the top of the organization and have self-renewal capacity. These cells give rise to their group of cells and differentiate into ST-HSC and multipotent progenitor cells (MPP). ST-HSC and MPP have limited self-renewal capacity and differentiate to lineage-specific progenitors. HSC are well studied stem cells as these cells have been under research for decades. They are studied extensively *in vitro* and *in vivo*, and are isolated and tested for therapeutic uses as well. Genetic engineering tools like genetic labeling and barcoding have also enhanced the study of HSC *in vivo* conditions. These studies are helpful to understand HSC and progenitor cell differentiation and self-renewal. Initial studies were conducted extensively in mice. Weissman and his team proved that mouse HSC can reconstitute blood components in irradiated mice (Spangrude, Heimfeld, and Weissman, 1988).

Hematopoiesis, the process of blood cell formation, begins during embryonic development. Following the formation of the three germ layers during gastrulation, blood cells originate from a specialized group of cells called hemangioblasts derived from the mesoderm (Ferretti and Hadjantonakis, 2019). Hemangioblasts are multipotent cells that can differentiate into both blood and endothelial cells. Around day 16–17 of human embryonic development, these specialized cells arise within the mesoderm layer of the yolk sac (Lacaud and Kouskoff, 2017). The process of hematopoiesis thus starts when cells aggregate and form structures known as blood islands. The hemangioblasts differentiate into two primary lineages: angioblasts, which contribute to the formation of blood vessels within the embryo, including the primitive vascular network in the yolk sac itself, and HSC, which give rise to blood cells. As embryonic development progresses, HSC within the yolk sac undergo expansion and multiplication, increasing their numbers and leading to the generation of blood islands (Padrón-Barthe et al., 2014).

Eventually, HSC migrate from the yolk sac to other organs involved in hematopoiesis, particularly the fetal liver and spleen (Gao et al., 2018). These organs provide a supportive environment for further differentiation and maturation of HSC. In these organs, HSC continue to differentiate and expand, producing the various types of blood cells. The fetal liver serves as the primary site for hematopoiesis during mid-gestation, while the spleen takes on this role during the later stages of embryonic development. Eventually, the bone marrow becomes the primary site for hematopoiesis in postnatal life. HSC colonize the bone marrow and establish a specialized microenvironment, known as the hematopoietic niche, where they can self-renew and differentiate into different blood cell lineages throughout an individual’s lifetime (Birbrair and Frenette, 2016). Therefore, different stages of development and locations within the body contribute to producing specific blood cell types.

A mouse model was developed using inducible sleeping beauty transposons and performed labeling and *in vivo* clone tracing (J. Sun et al., 2014). These studies demonstrated that more than LT-HSC, MPP play a significant role in maintaining a steady state. This observation was further confirmed by labelling studies in mice (Busch et al., 2015). They found that the lifetime of mice may not be good enough to study the equilibrium between labeled HSC and their progeny. Further studies with improved genetic engineering tools demonstrated the role of HSC in adult hematopoiesis (Sawai et al., 2016; Chapple et al., 2018).

Hematopoiesis is complex and tightly regulated by various signaling molecules and transcription factors (Edginton-White and Bonifer, 2022). Some key signaling pathways in this process work synergistically to regulate the specification, proliferation, and differentiation of cells involved in hematopoiesis. Notably, the NODAL/SMAD signaling pathway plays a crucial role in mesoderm formation during gastrulation (Wei and Wang, 2018). NODAL, a member of the transforming growth factor-beta (TGF-β) family, activates downstream signaling through SMAD proteins, promoting the specification of mesodermal cells. In parallel, WNT/β-catenin signaling regulates gene expression that supports mesoderm specification and subsequent differentiation into hemangioblasts (Tran et al., 2010). Additionally, Notch signaling is involved in specifying and maintaining hematopoietic stem/progenitor cells (Pajcini, Speck, and Pear, 2011). Activation of this pathway regulates the balance between self-renewal and differentiation of hemangioblasts, promoting their commitment to the hematopoietic lineage. Before that, vascular endothelial growth factor (VEGF) signaling is essential for developing blood vessels and forming blood islands within the yolk sac (Haigh, 2008). VEGF, acting through its receptors (VEGFR1 and VEGFR2), promotes the differentiation of angioblasts from the hemangioblast population. Angioblasts then contribute to forming the primitive vascular network within the yolk sac. Sonic Hedgehog (SHH) signaling is also involved in various aspects of embryonic development, including those that give rise to hemangioblasts (Davison and Zolessi, 2022).

These signaling events regulate transcription factors and chromatin components that work together to shape the gene regulatory networks controlling gene expression in the hematopoietic system and drive blood cell differentiation. For example, the GATA family of transcription factors plays an essential role in hematopoietic development (Lentjes et al., 2016). Likewise, HOX genes are crucial for establishing positional identity along the anterior-posterior axis during development, and specific HOX genes are involved in regulating the differentiation of hematopoietic stem cells and specifying lineage commitment of blood cells (Seifert, 2015). Finally, Runt-related transcription factor 1 (RUNX1) is essential for developing definitive hematopoiesis (Yzaguirre, de Bruijn, and Speck, 2017).

Increasing knowledge on HSC biology will significantly benefit the therapeutic use of these cells. Being able to accurately identify functional HSC and distinguish them from their heterogenous compartments has immensely facilitated their isolation. Also, uncovering the developmental journey of HSC has provided insight into the necessary cues and interactions that regulate their behavior, facilitating their manipulation *in vitro*. Nevertheless, mapping of individual hematopoietic cellular pathways with enough resolution is still undergoing, whose completion would be a giant leap towards unlocking the full potential of HSC *ex vivo* expansion.

## 4 Depicting HSC signaling towards cell expansion

### 4.1 Cytokines and growth factors

Cytokines and growth factors play a critical role in the expansion and maintenance of HSC, providing essential signals that regulate HSC self-renewal, proliferation, survival, and differentiation.

Stem cell factor (SCF), also known as c-kit ligand or kit ligand, is an early acting cytokine that is crucial in regulating HSC expansion and differentiation. It acts primarily through binding to its receptor, c-kit (CD117), which is expressed on the surface of HSC and various other HPC. The interaction between SCF and c-kit receptor triggers signaling pathways essential for the maintenance, proliferation, and survival of HSC. One of the critical pathways involved is the PI3K-Akt signaling pathway, which plays a central role in mediating the effects of SCF on HSC expansion (Cao et al., 2020). SCF can also activate other signaling pathways, such as the Ras-MAPK and the JAK-STAT pathways, which contribute to different aspects of HSC regulation, including survival, differentiation, and migration (Robinson et al., 2017).

Flt-3 Ligand (Flt-3L) is also an early acting growth factor that significantly regulates hematopoiesis, particularly in developing dendritic cells and lymphoid progenitors (Williams et al., 2017). Flt-3L has been shown to have some impact on HSC regulation. Flt-3L acts through its receptor, Flt3 (CD135), and activates both JAK-STAT and Ras-MAPK pathways. Concerning expansion of HSC and HPC, Flt-3L was included early on in studies on cytokine impact during HSC proliferation, often in combination with SCF given their synergistic role during the very early stages of human hematopoiesis. Optimization studies immediately determined a positive effect when including Flt-3L, contributing towards its recognition as a core growth factor in cytokine cocktails (Petzer et al., 1996; Andrade et al., 2010; Branco et al., 2020).

Unlike abovementioned cytokines, thrombopoietin (TPO) was initially found to be crucial in regulating dormancy of HSC in the bone marrow niche (Qian et al., 2007), while also regulating megakaryopoiesis and platelet production (de Sauvage et al., 1994; Decker et al., 2018). TPO primarily acts through its receptor, c-Mpl (Ninos et al., 2006), which is expressed on HSC and progenitor cells and triggers the activation of several signaling pathways that are important for HSC function, including JAK-STAT (Wilmes et al., 2020), PI3K-Akt (Jung et al., 2011), and Ras-MAPK (Majka et al., 2002). Its role in promoting HSC and HPC expansion has been uncovered when used with neonatal primitive cell populations, demonstrating its dual impact concerning proliferation. This has led to the incorporation of TPO as a decisive cytokine in UCB-derived HSC and HPC expansion protocols, showing synergistic effects with SCF and Flt-3L (Piacibello et al., 1997; Piacibello et al., 1998; McGuckin et al., 2004; Costa et al., 2018).

As with TPO, interleukin-6 (IL-6) is a multifunctional cytokine. Specifically, IL-6 plays a significant role in inflammation, immune response, and hematopoiesis. IL-6 can influence HSC behavior by interacting with specific receptors and activating downstream signaling pathways, such as the JAK-STAT pathway (Xiao et al., 2017). It is important to note that the influence of IL-6 on hematopoiesis can vary depending on factors such as the presence of other cytokines, the microenvironment, and the specific subset of HSC being targeted (Tie et al., 2019). Moreover, IL-6 has been shown to enhance the maintenance and proliferative capacity of primitive HSC in response to hypoxia in culture, without negatively impacting less primitive stem/progenitor cell populations (Duchez et al., 2015). HSC behavior can also be influenced by the cytokine dose. Specifically, IL-3 can stimulate HSC proliferation under low dosages (≤0.5 ng/mL), while high doses will strongly induce HSC differentiation (Ivanovic et al., 2011).

Finally, G-CSF is a cytokine that plays a critical role in regulating the production and differentiation of granulocytes. While the primary function of G-CSF is to stimulate the proliferation and differentiation of granulocyte precursors, it can indirectly affect HSC behavior as well. G-CSF acts through its receptor, expressed on the surface of various hematopoietic cells, including HSC and myeloid progenitors, and activates the JAK-STAT Pathway (Mui et al., 1995).

### 4.2 Cell cycle regulators

Cell cycle regulators are essential for maintaining the balance between HSC self-renewal and differentiation, controlling the progression through the different phases of the cell cycle, ensuring proper cell division, and maintaining the HSC pool. Cyclins are a group of proteins that play a crucial role in regulating the cell cycle. They control the progression of cells through different phases of the cell cycle, including G1, S, G2, and M phases (Kozar et al., 2004). While cyclins are not typically considered factors that directly regulate HSC expansion, they are essential components of the cell cycle machinery that can influence the proliferation and differentiation of stem and progenitor cells, including HSC. Cyclins interact with cyclin-dependent kinases (CDK), forming complexes that drive cell cycle progression. The activity of cyclin-CDK complexes is tightly regulated, and dysregulation can impact cell proliferation and differentiation. For example, cyclin D complexes with CDK4/6 regulate the G1 to S phase transition, cyclin E-CDK2 controls the G1-S transition, and cyclin A and cyclin B are involved in G2 and M phases, respectively. CDKs are a family of serine/threonine kinases that interact with cyclins to regulate cell cycle progression. CDK4 and CDK6, in association with Cyclin D, promote the G1 to S phase transition (Laurenti et al., 2015), while CDK2, in association with Cyclin E, drives cells from G1 to S phase (Aleem, Kiyokawa, and Kaldis, 2005). Retinoblastoma Protein (RB) is a tumor suppressor protein that regulates the G1 to S phase transition (Walkley and Orkin, 2006). RB binds to and inhibits the activity of E2F transcription factors, essential for the transcription of genes required for cell cycle progression (Burke et al., 2014). Phosphorylation of RB by CDKs relieves this inhibition, allowing cells to progress through the cell cycle. Checkpoint kinases, such as CHK1 and CHK2, are activated in response to DNA damage or replication stress. They play critical roles in cell cycle checkpoint control, halting cell cycle progression to allow for DNA repair and maintenance of genomic stability (Schuler et al., 2019).

### 4.3 Transcription factors and epigenetic modifiers

Transcription factors and epigenetic modifiers are crucial in regulating HSC function, self-renewal, lineage specification, and expansion. They control gene expression patterns and chromatin structure, influencing HSC fate decisions. GATA Binding Protein 2 (GATA2) is a transcription factor essential for the maintenance and expansion of HSC. It regulates the expression of critical HSC-associated genes, including SCF receptor, c-Kit, and Flt3, and is involved in HSC self-renewal and lineage commitment. GATA2 expression peaked in HSC and multipotent progenitors and diminished in committed myeloid progenitor cells and almost absent in terminally differentiated blood cell populations (Gonzalez et al., 2019). RUNX1 is a critical transcription factor for definitive hematopoiesis and HSC emergence during embryonic development (de Bruijn and Dzierzak, 2017; Valli et al., 2018). It regulates the balance between HSC self-renewal and differentiation and is involved in the transcriptional regulation of vital HSC-associated genes. Friend Leukemia Integration 1 (FLI1) plays a role in HSC self-renewal and lineage commitment. It regulates genes involved in hematopoietic cell proliferation, differentiation, and migration. FLI1 is essential for normal hematopoietic development (Giraud et al., 2021) and has been implicated in various hematological disorders, including acute myeloid leukemia (AML) (Kornblau et al., 2011), myelodysplastic syndrome (MDS) (Sullivan, Palmer, and Botero, 2022), and Ewing’s sarcoma (Hughes et al., 2023). It can act as a transcriptional activator and repressor, impacting gene expression in hematopoietic cells. Myeloid/Lymphoid or Mixed-Lineage Leukemia (MLL) is an epigenetic modifier that regulates HSC self-renewal and lineage specification through its histone methyltransferase activity, responsible for adding methyl groups to histone proteins, thereby affecting chromatin structure and gene expression. It controls the expression of critical HSC-associated genes, including Hox genes (Yu et al., 1998). While MLL is not a factor that directly regulates HSC expansion, its altered function can profoundly affect HSC behavior and hematopoiesis. Enhancer of Zeste Homolog 2 (EZH2) is a Polycomb Repressive Complex 2 (PRC2) component and catalyzes histone methylation, leading to gene silencing. EZH2 contributes to epigenetic modifications by catalyzing the methylation of histone H3 at lysine 27 (H3K27), leading to transcriptional repression (Herviou et al., 2016). It maintains a repressive chromatin state at specific genes, which can impact HSC function (Xie et al., 2014).

### 4.4 Metabolic regulators

Metabolic regulators play a pivotal role in regulating HSC function and maintenance and are involved in coordinating cellular energy metabolism, nutrient utilization, and stress responses.

Mammalian Target of Rapamycin (mTOR) is a protein kinase that is a central regulator of cellular growth, metabolism, and proliferation (Ling et al., 2023). It is crucial in integrating various signals, including nutrient availability and growth factors, to control cell behavior. mTOR also plays a role in regulating cellular metabolism, including nutrient utilization and energy production. This can indirectly affect HSC behavior by providing the necessary resources for expansion and maintenance. Hypoxia-inducible factors (HIFs) are transcription factors that respond to changes in oxygen levels. HIFs are heterodimeric complexes with an oxygen-sensitive HIF-α and a constitutively expressed beta subunit (HIF-β or ARNT). When oxygen levels are low (<5%) (hypoxia), HIF-α stabilizes and translocates to the nucleus, where it binds to specific DNA sequences to regulate the expression of target genes. HIF activation in HSC promotes glycolysis and suppresses mitochondrial respiration, maintaining HSC quiescence and self-renewal capacity under hypoxic conditions (Papandreou et al., 2006; Jakubison et al., 2022).

Amp-activated protein Kinase (AMPK) is a cellular energy sensor that plays a crucial role in maintaining energy homeostasis by regulating various metabolic processes. AMPK is activated under conditions of cellular energy stress, such as low levels of ATP and high levels of AMP. It can modulate glucose uptake, fatty acid oxidation, and mitochondrial biogenesis, among other processes. By regulating energy metabolism, AMPK can influence the availability of nutrients required for HSC expansion and maintenance (Saito and Nakada, 2014; Jacquel et al., 2018). Sirtuin proteins (SIRTs) are a family of NAD^+^-dependent deacetylases that regulate cellular metabolism and stress responses (Houtkooper, Pirinen, and Auwerx, 2012). SIRT1, in particular, is involved in HSC maintenance and self-renewal by modulating oxidative metabolism and DNA damage response (Z. Wang et al., 2022; Abraham et al., 2019).

Peroxisome proliferator-activated receptor gamma coactivator 1-alpha (PGC-1α) is a transcriptional coactivator that regulates mitochondrial biogenesis and oxidative metabolism (Basu, Broxmeyer, and Hangoc, 2013). It is essential for maintaining HSC function and quiescence by regulating mitochondrial activity and redox balance (Filippi and Ghaffari, 2019). These metabolic regulators influence HSC behavior by modulating vital metabolic pathways such as glycolysis, oxidative phosphorylation, fatty acid oxidation, and antioxidant defenses. They balance energy production and preservation, ensuring HSC quiescence, self-renewal, and long-term hematopoiesis.

## 5 Metabolic regulation of HSC

The metabolism and energy requirements of HSC play a crucial role in maintaining their self-renewal capacity, differentiation potential, and overall functionality. Within the bone marrow, HSC is predominantly quiescent or dormant, characterized by low metabolic activity. This quiescent state helps preserve the long-term self-renewal capacity of HSC and protects them from oxidative stress and DNA damage. During quiescence, HSC rely on anaerobic glycolysis as the primary energy source, converting glucose into pyruvate to generate a limited amount of ATP. HSC, particularly in the quiescent state, rely on glycolysis as their main energy-generating pathway (Simsek et al., 2010). Although quiescent HSC primarily rely on glycolysis, they do possess functional mitochondria. As HSC become activated, mitochondrial metabolism increases, and oxidative phosphorylation (OXPHOS) contributes more significantly to ATP production (Suda, Takubo, and Semenza, 2011). Fatty acid oxidation (FAO) is another energy-producing pathway used by HSC. As HSC exit quiescence and undergo activation and differentiation, they increase their reliance on FAO to meet their energy demands, involving the breakdown of fatty acids into acetyl-CoA that enter the tricarboxylic acid (TCA) cycle and lead to ATP production in mitochondria (Baldwin and Krebs, 1981). The metabolic activity of active HSC can lead to the generation of reactive oxygen species (ROS) as a by-product, having both detrimental and beneficial effects on HSC function (Giacomello et al., 2020). Excessive ROS can induce oxidative stress and damage DNA, while controlled levels of ROS are also crucial for HSC signaling. Overall, the metabolic state of HSC is dynamic and varies depending on their quiescent or activated state. Understanding the intricacies of HSC metabolism is crucial for elucidating their biology and developing strategies to manipulate HSC function for therapeutic purposes.

Several signaling pathways play essential roles in regulating HSC metabolism. These pathways contribute to maintaining HSC quiescence, self-renewal, and differentiation.

The HIF pathway is activated in response to low oxygen levels and plays a crucial role in adapting cellular metabolism to cope with changing oxygen conditions. Throughout adulthood, HSC reside in a specialized microenvironment within the bone marrow, where oxygen levels can fluctuate due to factors like cell density and blood flow (Eliasson and Jönsson, 2010a). Understanding the correlation between the HIF pathway and HSC metabolism is pivotal for deciphering how HSC maintain their functionality under changing oxygen concentrations. Under hypoxic conditions in the bone marrow (with gradients between <1% and 6%), HSC upregulate glycolysis (Eliasson and Jönsson, 2010b; Kocabas et al., 2015). HIF activation can suppress mitochondrial function, particularly oxidative metabolism (Huang et al., 2022). This reduction in mitochondrial respiration is thought to contribute to maintaining HSC quiescence and preventing excessive oxidative stress, which could otherwise be detrimental to stem cell functionality. mTOR and Akt, central to the path, modulate cellular metabolism, including glucose uptake and mitochondrial function, influencing HSC energy production, quiescence, and functionality (Saxton and Sabatini, 2017). There is an intricate interplay between the PI3K-Akt-mTOR pathway and the HIF pathway. Akt can directly stabilize HIF-1α, a vital component of the HIF pathway, under normoxic (i.e., atmospheric air) conditions (Düvel et al., 2010).

The AMPK pathway acts as an energy sensor and master regulator of cellular energy homeostasis. This pathway dynamically interfaces with other pivotal signaling cascades to harmonize metabolic equilibrium and ensure the functionality of HSC. The AMPK and PI3K-Akt-mTOR pathways form an intricate seesaw, where one inhibits while the other activates (Saxton and Sabatini, 2017). AMPK activation restricts mTORC1 activity, stifling energy-intensive processes such as protein synthesis and promoting catabolic pathways like autophagy. In contrast, the mTORC1 pathway suppresses AMPK activity. HSC also treads the interplay between the AMPK and HIF pathways (Moldogazieva, Mokhosoev, and Terentiev, 2020). Activation of AMPK during energy deprivation favors HIF-1α degradation under normoxic conditions, fine-tuning HIF-dependent metabolic adaptations in response to hypoxia. The AMPK pathway can modulate the Wnt/β-catenin pathway, a crucial determinant of stem cell fate (Zhao et al., 2010). AMPK activation attenuates Wnt/β-catenin signaling, potentially influencing HSC self-renewal and differentiation choices. The activation of the Wnt/β-catenin signaling enhances glycolytic metabolism and ATP production (Ouwens et al., 2022), thus promoting HSC proliferation.

The Peroxisome Proliferator-Activated Receptor Gamma (PPARγ) pathway intricately intersects with the signaling pathways governing metabolism, contributing to the finely tuned orchestration of cellular energy balance and metabolic homeostasis (K. Ito et al., 2012). PPARγ and the AMPK pathway share a reciprocal connection (Q. Wang et al., 2021). AMPK activation can enhance PPARγ activity, promoting adipogenesis and lipid metabolism. Conversely, PPARγ activation can influence AMPK-mediated processes, modulating cellular energy utilization and enabling glucose uptake. The PPARγ and Wnt/β-catenin pathways often exhibit reciprocal activity (Lecarpentier et al., 2017). PPARγ activation can inhibit Wnt signaling, and conversely, Wnt signaling can suppress PPARγ expression, influencing lipid metabolism and adipogenesis. The mTOR pathway can also intersect with the PPARγ pathway (Souza-Moreira et al., 2019). mTOR controls adipogenesis and lipid metabolism, which are also governed by PPARγ. PPARγ activation can impact HIF-1α stability, potentially affecting HIF-dependent metabolic adaptations to hypoxia (Blum et al., 2016). Additionally, PPARγ activation influences insulin sensitivity and glucose metabolism (Leonardini et al., 2009).

## 6 *Ex Vivo* expansion of HSC and HPC

Generating desired numbers of HSC and HPC *in vitro* is a tremendous feat. Harnessing the expansion potential of hematopoietic cells is meaningful, whose turnover is an impressive 90% of the total turnover of a human body (i.e., 0.33 ± 0.02 × 10^12^ cells per day) (Sender and Milo, 2021). Development and rapid evolution of *ex vivo* expansion of HSC and HPC have been pushed essentially by researchers and clinicians tackling the limited application of UCB units as a cell source for HCT. The therapeutic potential of expanded HSC and HPC was observed early on, with innovative studies demonstrating their safety and feasibility, as well as their ability to alleviate post-myeloablative neutropenia (Alcorn et al., 1996; Reiffers et al., 1999). Nevertheless, new fields of study also take advantage of possibly having HSC and HPC as an unlimited raw material, such as hematopoietic gene therapy, erythrocyte production for transfusions, and hematological disease modeling (Tucci et al., 2021; Pellegrin, Severn, and Toye, 2021; filho and Ghaffari, 2019).

**FIGURE 2:**
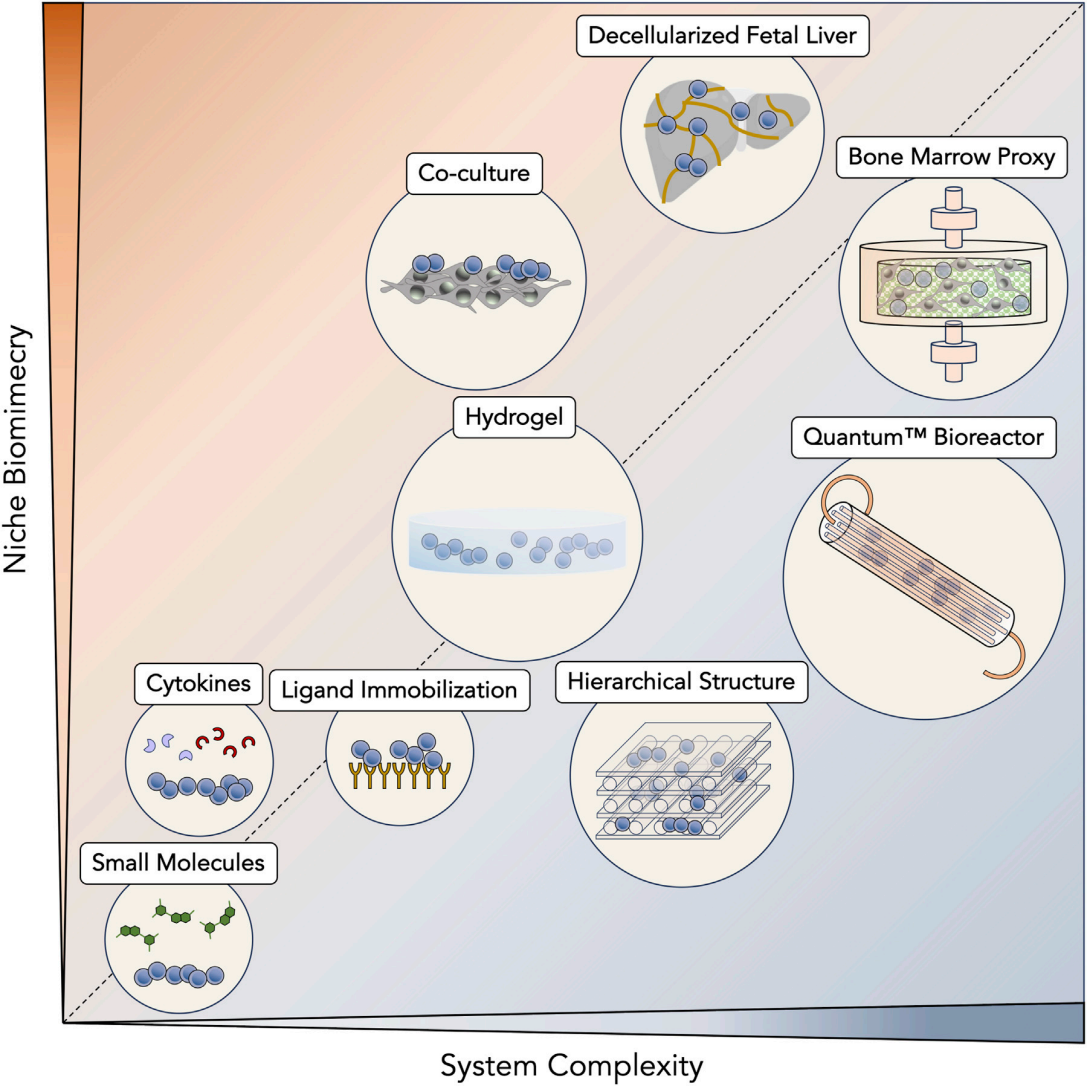
Diversity of *ex vivo* expansion strategies for hematopoietic stem cells (HSC) and progenitor cells (HPC). Expansion systems have been scaled according to their intrinsic complexity as well as their ability to mimic hematopoietic niches. Simpler systems, such as those that are based on supplementation of small molecules and cytokines, benefit from an easier translation to a manufacturing scenario but are unable to reconstitute most interactions from native primitive cell niches. Still, between them, cytokines are only ones to be naturally present in hematopoietic niches. Immobilization of ligands taps into similar established pathways of the niche (e.g., Notch signaling pathway), but represents a step up in complexity due to their necessary immobilization in expansion platforms. The use of hydrogels brings a significant impact on stem/progenitor cell surroundings, being able to recreate a three-dimensional (3D) environment. These materials are able to fine-tune more complex volumetric variables, such as stiffness and 3D porosity. In another approach, incorporation of other cell types in co-culture systems enhances the natural cellular interactions that are present in hematopoietic niches and that contribute to their *in vivo* regulation. Biomaterial-based systems have brought considerable versatility to their design and are able to mimic native niches at a desired level, ultimately reaching synthetic bone marrow structures. Going for more controlled systems that are compatible with real-time monitoring, bioreactors are the epitome of artificial systems that are most aligned with large-scale bioprocessing and clinical-grade production. However, they are typically resistant with the integration of more complex biological structures, such as the bone marrow. Contrarily, decellularization approaches aim to take advantage of the native niches themselves, with their intrinsic complexity and architecture. This strategy fully respects biomimicry of hematopoietic niches and their potential to induce the expansion of HSC and HPC.

Initial protocols for HSC and HPC expansion were solely based on cytokine supplementation, causing a chase for novel ligands or signaling molecules responsible for promoting self-renewal. At that time, along with basal medium formulations and animal serum, cytokines and growth factors became the bedrock of any expansion system. While a great variety of cytokines were sequentially discovered to promote stem/progenitor expansion, a recurrent core of signaling molecules, including SCF, Flt-3L, TPO and IL-6, have solidified their almost ubiquitous presence in expansion cultures (early protocols—reviewed in (Lund et al., 2015; Pineault and Abu-Khader, 2015; Costa et al., 2018); recent protocols—Table 1). Improvement of cytokine-based expansion then turned to the development of tailored expansion media, specifically tuned to improve proliferation of HSC and HPC (Qiu et al., 1999; Allison et al., 2004; Sei et al., 2019). Beyond personalizing basal formulations, undefined sera (e.g., fetal bovine serum (FBS)) were phased out, significantly reducing batch variability during expansions. In the hope of eventually reaching chemically defined media, remaining animal-derived products have also been slowly substituted, coalescing with recommendations by regulatory agencies for current good manufacturing practices (cGMP) (Lipsitz, Timmins, and Zandstra, 2016). As cytokine-based expansion became more widespread, to boost the proliferation of HSC and HPC further, cytokine concentrations were identified as a relevant target for optimization. Fine-tuning cytokine concentrations was achieved through experimental design, uncovering synergistic relationships and giving rise to multiple optimized cytokine cocktails (Pineault et al., 2011; Branco et al., 2020). Although cytokine-based expansion alone could expand HSC and HPC enough to support numerous clinical trials (Shpall et al., 2002; Kiernan et al., 2017), numerous strategies have now evolved from this initial platform (Figure 2).

**TABLE 1:** Overview of the diversity of relevant strategies for *ex vivo* expansion of hematopoietic stem and progenitor cells.

System	System description	Media	Cytokine combination	Duration of culture	Expansion fold	Clinical trials	References
Co-culture with MSC	UCB MNC were placed in co-culture with MSC STRO-3^+^ feeder layer-containing flasks. After 7 days, non-adherent fraction is transferred to culture bags and flasks are replenished with fresh medium and cultured for an additional 14 days	GMP-grade serum-free medium	SCF, FLT-3L, TPO and GCSF	14 days (Several medium changes)	Median FC TNC = 12.2	NCT00498316NCT03096782	Lima et al. (2012)
					Median FC CD34^+^ = 30.1		
					FC CFU-C = 17.5		
Co-culture with MSC	UCB CD34^+^ cells are co-cultured with BM/UCM/AT MSC feeder layers	StemSpan SFEM II	Optimized Cocktail SCF, Flt-3L, TPO and bFGF	7 days (No medium changes)	Mean FC TNC =	—	Branco et al. (2020); Bucar et al. (2021)
					BM: 74; UCM: 50; AT: 83		
					Mean FC CD34^+^ =		
					BM: 52; UCM: 23; AT: 57		
Co-culture with engineered endothelial cells	Genetically modified primary HUVEC were co-cultured with UCB CD34^+^ cells. When expanded hematopoietic cell density reached 2 × 10^6^ cells/mL, non-adherent cells were transferred to a fresh endothelial cell feeder layer and fresh medium	X-Vivo 20/StemSpan	SCF, Flt-3L and TPO	12 days (Medium changes every other day)	Mean FC TNC = 400	NCT03483324	Butler et al. (2012)
					Mean FC CD34^+^ = 150		
					Mean FC Lin^-^CD34^+^CD45RA^-^CD49f^+^ = 23		
Notch-ligand	UCB CD34^+^ cells are expanded in cultureware coated with Delta1-ext^IgG^	StemSpan SFEM	SCF, Flt-3L, TPO, IL-6 and IL-3	16 days (Medium changes every 3–4 days)	Mean FC TNC = 562Mean FC CD34^+^ = 164FC SRC frequency = 16	NCT01031368NCT03301597NCT00343798NCT04083170NCT01175785NCT03399773NCT01690520	Delaney et al. (2010); Milano et al. (2022)
Hypoxia w/notch ligand	Mobilized PB CD34^+^ were expanded in hypoxia (1%–2% oxygen) with Delta1-ext^IgG^ coatings	StemSpan SFEM II	SCF, Flt-3L and TPO	21 days (Medium changes every 3 days)	Mean FC TNC = 50	—	Araki et al. (2021)
					Mean FC CD34^+^ = 18		
Membrane bioreactor (Quantum™)	UCB CD34^+^ cells are expanded in the lumen of a Quantum™ bioreactor with pre-coating of fibronectin and SDF-1	GMP serum-free SCGM medium (CellGenix)	SCF, Flt-3L, TPO, IL-3, IL-6, GDNF and SR-1	8 days (Perfusion—from 0.1 to 0.2 mL/min)	Mean FC CD34^+^ = 51	—	Jones et al. (2022)
Zwitterionic hydrogel	UCB CD34^+^ were expanded in a zwitterionic hydrogel	StemSpan SFEM II	SCF, Flt-3L, TPO, IL-6 and IL-3	24 days (Added Fresh Medium - 10% culture volume every day)	Mean FC TNC = 322	—	Bai et al. (2019)
					Mean FC CD34^+^ = 319		
					Mean FC CD34^+^CD38^-^CD45RA^-^CD49f^+^CD90^+^ = 284		
Bone marrow proxy	UCB CD34^+^ cells were expanded in a biomimetic hydroxyapatite scaffold previously populated by osteogenesis-primed MSC in a perfusion bioreactor.	StemSpan SFEM	SCF, Flt-3L and TPO	7 days (Cyclic Perfusion - 280 uL/s; Medium change twice a week)	Mean FC TNC = 61	—	Bourgine et al. (2018)
					Mean FC CD34^+^CD38^-^ = 45		
					Mean FC CD34^+^CD38^-^CD45RA^-^CD90^+^ = 13		
Extracellular vesicles	UCB CD34^+^ cells were expanded with modulated and native osteoblast-derived extracellular vesicles	Cellgenix GMP SCGM medium	SCF and Flt-3L	10 days (Medium change every 2–3 days)	Mean FC TNC = 13.4	—	Morhayim et al. (2020)
					Mean FC CD34^+^ = 7.8		
Chemically-defined medium	UCB CD34^+^ cells were expanded without exogenous cytokines and with a chemically-defined medium.	IMDM	740Y-P, butyzamide, PCL-PVAc-PEG and UM171	30 days (Medium change every 3 days)	UCB:	—	Sakurai et al. (2023)
					Mean FC TNC = 75		
					Mean FC CD34^+^ = 55		
					PB:		
					Mean FC TNC = 9		
dECM matrices	UCB CD34^+^ were expanded on spin coated decellularized extracellular matrix from HS-5 cells (human fibroblast-like cell line)	Stemline	SCF, TPO, IL-6 and Flt-3L	8 days (No medium change)	Mean FC TNC = 105	—	Wasnik et al. (2016)
					Mean FC CD34^+^CD38^-^ = 12		
3D hierarchical scaffolds	UCB CD34^+^ were expanded on a hierarchical scaffold of lattices made of 10% hydroxyapatite in polycaprolactone and a fibrous mesh made up of polyuretane coated with vitronectin. Cells were expanded in intermittent hydrostatic pressure	StemSpan SFEM II	SCF, Flt-3L and TPO	7 days (Medium change every other day)	Mean FC TNC = 21	—	Kim et al. (2019)
					Mean FC CD34^+^ = 11		
3D hydrogel	Circulating stem/progenitor cells from PB were expanded in polypeptide hydrogels	StemSpan SFEM II	SCF, FLT-3L, TPO, VEGF, IL-3, IL-6, SR-1 and Vitamin C	14 days	Mean FC CD34^+^ = 70	—	Xu et al. (2022)
Fetal liver construct	UCB CD34^+^ were expanded on a ferret-derived decellularized liver, after repopulation with human fetal liver stromal cells	QSBF-60 serum-free medium	SCF, Flt-3L, LIF and bFGF	12 days	Mean FC TNC = 380	—	Mokhtari et al. (2018)

### 6.1 EV-mediated strategies

The discovery of extracellular vesicles (EV) has disrupted our established knowledge of cell biology by uncovering new means of cell communication (Raposo and Stahl, 2019). Since mechanisms that benefit the expansion of hematopoietic stem/progenitor cells are largely unknown, EV have been proposed as a possible key player. EV may partially explain hematopoietic niche homeostasis maintained by differentiated endothelial cells (EC) or more primitive supportive cell types, namely, mesenchymal stromal cells (MSC). Naturally, MSC-derived EV were one of the initial candidates to be tested on HSC expansion settings (Xie et al., 2016; Ghebes et al., 2021). BM-derived MSC EV of fetal or adult origin were evaluated side-by-side for their possible hematopoietic supportive capacity (Ghebes et al., 2021). While EV isolated from adult BM MSC nearly doubled TNC fold change (FC) and CD34^+^ cell FC compared to an experimental control (i.e., expansion without EV), fetal EV showed no advantage compared to control conditions. Through proteomic profiling, insight into the EV cargo uncovered the enrichment of proteins involved in the TGF-β receptor pathway in EV from fetal BM MSC. TGF-β is described as one of the major inhibitors of HSC and HPC proliferation, and a recent report has also identified the presence of Smad2, a TGF-β signal transducer, in murine MSC-derived EV (Blank and Karlsson, 2015; Gautheron et al., 2023). Consistently, adding a TGFB1 inhibitor to the expansion cocktail, including fetal BM MSC-derived EV, significantly increased the expansion of TNC, CD34^+,^ and CD34^+^CD38^−^CD45RA^−^ expressing cells (Ghebes et al., 2021). Intriguingly, osteoblast-derived EV have been described to have a similar effect on HSC and HPC expansion (Morhayim et al., 2020). Although belonging to a quiescence-promoting microenvironment (i.e., endosteal niche), osteoblast-derived EV led to a 2.4 FC of CD34^+^ cells relative to increases without EV supplementation. Here, the most abundant elements of the EV cargo, both at the miRNA and protein level, were identified as having the *EGR1* gene as a target, which encodes a transcription factor responsible for HSC regulation (Morhayim et al., 2020). The use of EV is still in its infancy in what concerns HSC and HPC expansion, with a lack of standardized isolation protocols and robust characterization tools as relevant obstacles. As expected with these preliminary studies, exhaustive functional evaluation of expanded cells (e.g., *in vivo* repopulation assays) is lacking and should be included as the use of EV as a co-adjuvant for HSC and HPC expansion increases. Nevertheless, a higher expansion of cobblestone area-forming cell (CAFC) colonies (a proxy for more primitive hematopoietic populations) has been reported when MSC-derived EV were included in the expansion cocktail (Xie et al., 2016).

Improvement of an EV-based benefit for the expansion of HSC and HPC largely depends on cargo manipulation. A more native approach would explore other cell types as EV producers to find the ideal match between cargo and hematopoietic supportive properties. Another strategy has been considering EV as a cellular derivative, to apply cell pre-conditioning to have the producing cell alter its EV composition. By exposing MSC to a hypoxia culture (1% O_2_) before EV collection, changes in their native EV cargo were observed, which led to improved TNC FC and the percentage of CD133^+^ cells (Niazi et al., 2022). Still, the ultimate objective in the field in order to harness the full potential of EV as systems of signaling modulation would be to bioengineer EV to reach customizable cargos.

### 6.2 Small molecules

Recognizing the simplicity of supplementation of recombinant cytokines and growth factors in *ex vivo* expansion protocols, small molecules have also penetrated the field. Cytokines can be considerably expensive since their production model is based on cell factories using recombinant DNA technology with significant downstream purification involved (Yasuda et al., 2018). Also, due to the complexity of cell signaling, a single cytokine may have several functions or influence multiple pathways, resulting in unwanted off-target signaling (Spangler et al., 2015). Small molecules can be chemically manufactured, bypassing the biological production burden. While initially tallying only a few molecules, small molecules have become the most rapidly expanding category of stem/progenitor cell expansion promoters (Table 2). Still, several studies have demonstrated that the role of cytokines is irreplaceable and deemed necessary for HSC and HPC expansion *ex vivo*, and small molecules can modulate the ability of cytokines to enhance HSC stemness and proliferation (L. Wang et al., 2017). The suitability of employing small molecules in combination with different existing cytokine cocktails should be carefully assessed towards optimization of *ex vivo* primitive cell expansion. Going forward, mention of small molecules in this review implies their use along with different cytokines.

**TABLE 2:** Description of the mechanism of action of pre-clinical and clinically relevant small molecules for *ex vivo* expansion of hematopoietic stem and progenitor cells.

Small molecule	By inhibiting	By promoting	Known/possible mechanism	Clinical trials	References
UM171	Receptor		Inhibiting coREST complex	Yes	Fares et al., 2014; D. Kim, Kim, and Baek, 2021; Subramaniam et al., 2020; Cohen et al., 2020; Claveau et al., 2020
			Suppression of erythroid and megakaryocytic differentiation		
UM729	Pathway		Inhibiting alpha 4 beta 1 integrin pathway and promoting AhR	Yes	Fares et al. (2014)
PGE2 (FT1050)		Pathway	Activating EP4 receptor on HSC	Yes	Fehér and Gidáli, 1974; Gidáli and Fehėr, 1977; North et al., 2007; Hoggatt et al., 2008; Goessling et al., 2009
			Increasing in homing, survival and proliferation		
SW033291		PGE2	Inhibiting 15-PGDH, leading to increase in PGE2 levels		(Y. Zhang et al., 2015)
SW209415		PGE2	Inhibiting 15-PGDH, leading to increase in PGE2 levels		Desai et al. (2018)
GW9662		Mitochondrial metabolism	Antagonist of PPAR-γ; Inhibiting PPAR-γ 3 and promoting stem/progenitor cell expansion by regulating mitochondrial metabolism and decreasing HSC differentiation		Guo et al. (2018)
T0070907		Mitochondrial metabolism	Antagonist of PPAR-γ; Inhibiting PPAR-γ 3 and promoting stem/progenitor cell expansion by regulating mitochondrial metabolism and decreasing HSC differentiation		Guo et al. (2018)
MB05032		Mitochondrial metabolism	FBP-1 inhibitor; Promoting HSC expansion and inhibiting differentiation due to elevated glycolysis through the PPARG-FBP1 axis		Guo et al. (2018)
StemRegenin-1	Receptor		Promoting HSC expansion by inhibiting AhR	Yes	Boitano et al., 2010; Yin et al., 2016
DEAB	Metabolism		Inhibiting HSC differentiation via ALDH suppression, leading to downregulation of retinoic acid signaling pathway and overexpression of cEBP.		Chute et al., 2005 (2006), Cooper et al., 2018
Valproic Acid	HDAC		HDAC inhibitor; Overexpression of quiescence genes by histone acetylation	Yes	Bug et al., 2005; Felice et al., 2005; Seet et al., 2009; Walasek et al., 2012; Papa et al., 2018
Nicotinamide	Metabolism		Inhibiting SIRT1 and decreasing p21 expression	Yes	Peled et al., 2012; Campbell et al., 2019; Vannini et al., 2019
Rapamycin	Pathway		Inhibiting mTOR.		Huang et al. (2012)
CHIR99021	Pathway		Activating the Wnt pathway		Trowbridge et al., 2006; Huang et al., 2012; Li et al., 2019
SB203580	Apoptosis		Inhibiting apoptosis through p38 MAPK; decline in HSC senescence and overexpression of CXCR4		Bari et al. (2018)
TEPA	Histone Acetyl-transferases		Decreasing acetylation; Increasing the activity of cytochrome C oxidase	Yes	Treves, Landau, and Peled, 2002; Peled et al., 2004; Peled et al., 2005; Lima et al., 2008
zVADfmk	Apoptosis		Blocking apoptosis; Pan-Caspase inhibitor, leading to overexpression of Bcl-2, downregulation of Caspase-3 and activation of Notch 1		Sangeetha, Kale, and Limaye, (2010)
zLLYfmk	Apoptosis		Apoptotic protease and calpains inhibitor was shown to increase CD34^+^ cell content during *ex vivo* expansion		Sangeetha, Kale, and Limaye, (2010)
BIO	Pathway		GSK3 Inhibitor		Li et al. (2019)
CPI203	Bromodomain motifs		Bromodomain and extra-terminal motif inhibitor increasing LT-HSC		Hua et al. (2020)

#### 6.2.1 Clinically relevant molecules

**FIGURE 3:**
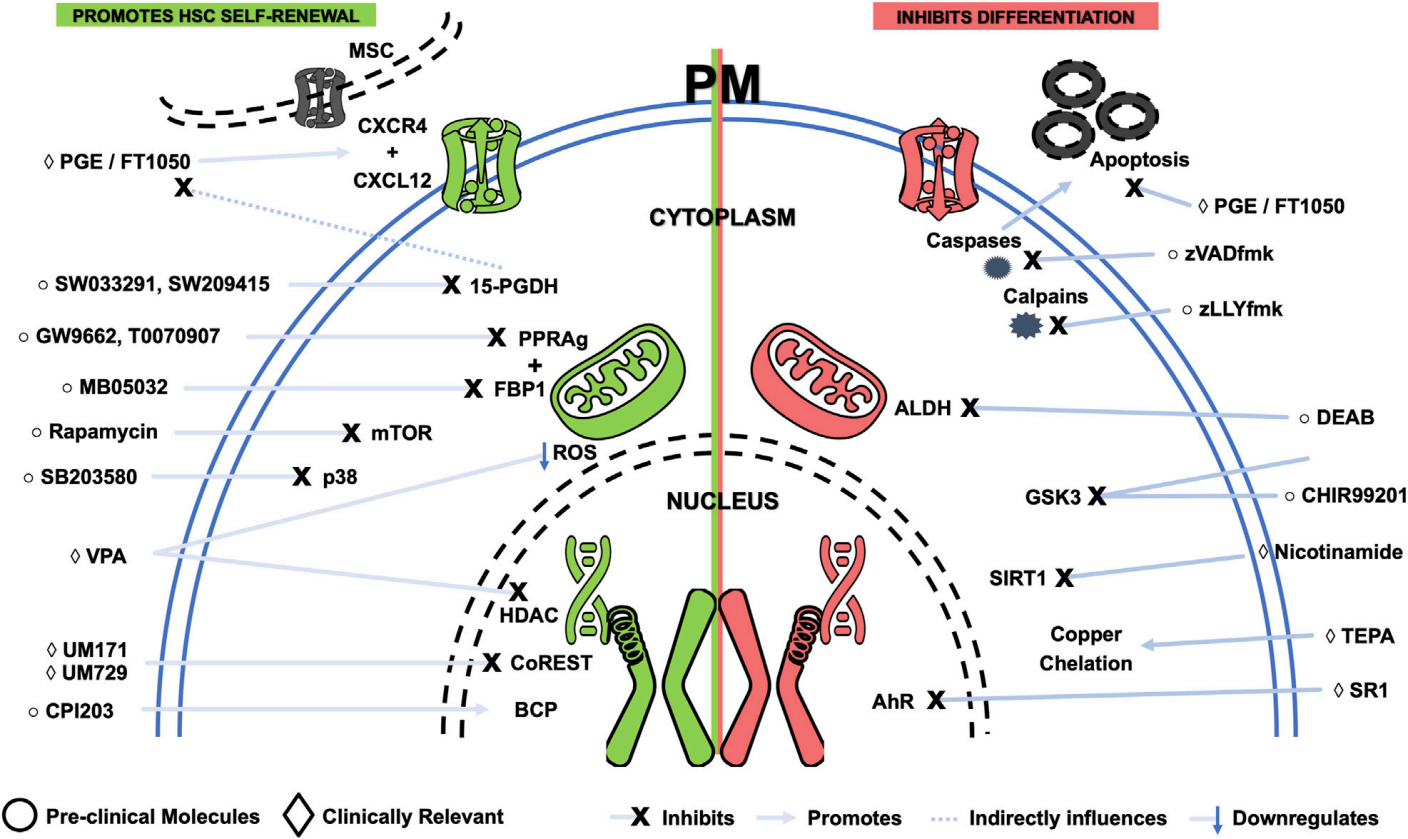
Schematic representing small molecules that benefit expansion of hematopoietic stem and progenitor cells, either by promoting self-renewal (green) or by inhibiting differentiation (red). Targets and simplified mechanisms of action are shown, with spatial discrimination between extracellular space, plasma membrane (PM), cytoplasm and nucleus. Pre-clinical molecules are identified by a circle symbol, while clinically relevant small molecules are tagged by a diamond symbol. AhR - Aryl hydrocarbon receptor; ALDH—Aldehyde dehydrogenase; BCP - Bromodomain-containing proteins; BIO - 6-bromoindirubin-3′-oxime; DEAB—Diethylaminobenzaldehyde; HDAC - Histone deacetylases; MSC—Mesenchymal stromal cell; mTOR - Mammalian target of rapamycin; PGE - Prostaglandin E; ROS—Reactive oxygen species; SR1—StemRegenin-1; TEPA—Tetraethylenepentamine; VPA—Valproic acid.

Due to their pre-clinical success in HSC/HPC expansion settings, various small molecules have made their way through the clinical pipeline toward regulatory approval. Although their mode of action (MoA) is typically unknown, knowledge about their impact on cellular mechanisms has been growing (Figure 3). This is essential when developing synthetic molecules to avoid undesired off-target effects.

UM729 is a small molecule that has been shown to promote the expansion of HSC *in vitro* (Fares et al., 2014). Identified through a high-throughput screen of 5,280 compounds, UM729 has been explicitly shown to enhance HSC self-renewal and increase functional HSC by up to 10-fold. Besides promoting HSC self-renewal, UM729 was also able to maintain the long-term repopulating capacity of HSC, which is essential for ensuring that expanded cells maintain their fundamental functions.

As a derivative of UM729, UM171 was discovered to be 10 to 20 times more potent in influencing the expansion of HSC *in vitro* and *in vivo* by considerably improving the expansion fold of CD34^+^CD38^−^ cells and their engraftment potential (Fares et al., 2014). UM171 has been described to target the CoREST complex, a protein complex that inhibits HSC self-renewal, and the JAK-STAT signaling pathway. Specifically, this small molecule has been shown to bind to the CoREST complex and recruit the E3 ligase CUL4A that catalyzes the degradation of LSD1 (a protein that is part of the CoREST complex). The degradation of LSD1 leads to the release of the repressed genes, which then promote HSC self-renewal (D. Kim, Kim, and Baek, 2021). In tandem, UM171 also activates the non-canonical Wnt signaling pathway, which protects cells from damage and promotes their survival (Subramaniam et al., 2020). UM171-based HSC and HPC expansion has been recently evaluated through a clinical trial for single-arm UCB HCT to treat hematological malignancies (Cohen et al., 2020). Successful application of UM171-based expanded cells for a non-malignant hematopoietic disease (i.e., aplastic anemia) has highlighted its potential use for other disorders (Claveau et al., 2020). Of note, UM171 and other small molecules have been reported to induce upregulation of HSC markers (e.g., CD90) without the accompanying cell functionality (i.e., repopulating activity). Thus, functional confirmation of cell identity is always needed to avoid the generation of non-functional cells (Goncalves et al., 2018).

Through modulation of the aryl hydrocarbon receptor (AhR) pathway, StemRegenin-1 (SR-1) was also identified as a small molecule of great interest, inhibiting this pathway (Boitano et al., 2010b). Also detected through high-throughput screening, SR-1 targets the AhR, a transcription factor involved in regulating genes involved in cell growth, differentiation, and apoptosis (Yin et al., 2016). When SR-1 binds to the AhR, it prevents it from binding to its DNA target sequences. This prevents the AhR from activating genes that promote cell growth and differentiation and activating apoptosis-promoting genes. SR-1 treatment significantly increased the number of CD34^+^CD38^−^ cells in human UCB cultures, while also increasing the frequency of long-term culture-initiating cells (LTC-IC) and colony-forming unit (CFU) (Boitano et al., 2010b). SR-1 also improved the survival of mice transplanted with SR-1 expanded HSC, with the number of HSC detected after transplantation increasing 100-fold. Consequently, SR-1 has been used to enhance the expansion of HSC and HPC in clinical trials assessing HCT. SR-1 was being evaluated in clinical trials for treating hematologic malignancies and inherited metabolic diseases. Although clinical development of SR-1 for inherited metabolic diseases was discontinued, a Phase II trial for high-risk hematological malignancies is currently active (NCT03674411).

Prostaglandin E2 (PGE2) is a hormone involved in various cellular processes, including cell growth, differentiation, and apoptosis, and has also been shown to have a stimulative effect on HSC and HPC (Fehér and Gidáli, 1974; Gidáli and Fehėr, 1977). By activating the Wnt signaling pathway, which regulates HSC self-renewal and differentiation, PGE2 can promote the expansion of stem/progenitor cell *in vitro* and *in vivo* (North et al., 2007; Hoggatt et al., 2008; Goessling et al., 2009). PGE2 has also been shown to protect HSC from apoptosis. Treatment with PGE2 has been described to increase the number of HSC by up to 4-fold while not affecting their differentiation potential. However, PGE2 is a hormone that plays a role in inflammation, pain, and fever. FT1050, a 16,16-dimethyl PGE2 (developed by Fate Therapeutics), has been designed to increase HSC number and function by activating key pathways that guide cell fate (Cutler et al., 2011). FT1050 is currently being explored in clinical trials for treating hematologic malignancies, such as leukemia and lymphoma. The mechanisms of action of FT1050 have been demonstrated to involve improved homing via the CXCL12-CXCR4 axis, increased proliferation and cell cycle entry, and decreased apoptosis rates (Desponts et al., 2010; Cutler et al., 2011; Cutler et al., 2014; Guerrettaz et al., 2015). After displaying success in an initial Phase Ib clinical trial (NCT01527838), FT1050 progressed to a Phase II trial (NCT01627314) in adult patients with hematologic malignancies who have undergone nonmyeloablative conditioning therapy. Unfortunately, Fate Therapeutics discontinued the clinical development of FT1050, albeit with supportive data from the abovementioned Phase II trial.

Nicotinamide is a form of vitamin B3 that has been shown to inhibit differentiation and facilitate the expansion of HSC and HPC with enhanced bone marrow homing and engraftment. The mechanism behind the impact of nicotinamide involves inhibition of SIRT1, a deacetylase. By influencing protein acetylation through SIRT1 inhibition, nicotinamide prevents HSC from differentiating into mature blood cells, allowing them to expand in culture (Peled et al., 2012). Interestingly, a recent study found that nicotinamide riboside (NR), a precursor of nicotinamide, can attenuate age-associated metabolic and functional changes in HSC (Sun et al., 2021). NR treatment was associated with improved engraftment and partial reversion of the myeloid bias, demonstrated in transplantation assays using HSC isolated from NR-treated aged mice. Besides being part of the portfolio of Gamida Cell, nicotinamide had also been incorporated early on in some serum-free media formulations at a very low concentration, demonstrating its long track record in clinical significance before regulatory approval (Peled et al., 2004; Ivanovic et al., 2006). Initially, due to the innovative nature of *ex vivo* expanded cord blood units in the clinical pipeline, expanded units were required to be clinically evaluated in combination with a non-expanded cord unit (Horwitz et al., 2014). After demonstrating their value in DUCBT, single-arm transplants using only the expanded unit were assessed (Horwitz et al., 2019). A recent Phase III clinical trial reached clear conclusions about using nicotinamide-based expansion for HCT, displaying improved neutrophil and platelet reconstitution and reduced bacterial, fungal, and viral infections (Horwitz et al., 2021). The results were pivotal in assisting nicotinamide-based expansion to reach regulatory approval by the United States Food & Drug Administration (FDA) (commercially known as Omisirge) and become the first approved cell therapy product based on *ex vivo* expanded HSC and HPC (“FDA Approves Cord-Blood Therapy” 2023).

One of the earliest pre-clinical candidates for HSC and HPC expansion, the epigenic modulator tetraethylenepentamine (TEPA), a copper chelator, has been shown to enhance HSC self-renewal by inhibiting HSC differentiation (Treves, Landau, and Peled, 2002; Peled et al., 2005). The identification of this small molecule was based on the knowledge of cell differentiation being augmented in the presence of high copper concentration. As a copper chelator, TEPA is able to reduce levels of copper ions, inhibiting unwanted differentiation. Treatment with TEPA showed a robust increase in UCB-derived stem/progenitor cells in culture, which showed repopulating capacity in NOD/SCID mice (T. Peled et al., 2004; Peled et al., 2004). After proving its safe use in a Phase I trial (Lima et al., 2008), TEPA-based expansion progressed to a multicenter Phase II/III clinical trial (NCT00469729), demonstrating faster neutrophil and platelet engraftment, as well as a 77-fold increase of CD34^+^ cells before patient infusion (P. J. Stiff et al., 2018). Also part of the portfolio of Gamida Cell, TEPA has not seen developments in its clinical progression in recent years, possibly due to an internal decision of Gamida Cell (Zimran, Papa, and Hoffman, 2021).

Valproic acid (VPA) is a histone deacetylase inhibitor shown to expand the number of HSC and HPC *in vitro*. VPA works by inhibiting histone acetyltransferases, which remove acetyl groups from histone proteins. Acetylation of histones loosens the structure of chromatin, which allows genes to be transcribed. Inhibition of these enzymes by VPA leads to increased gene expression in HSC, which promotes their proliferation and differentiation (Bug et al., 2005; Felice et al., 2005; Seet et al., 2009; Walasek et al., 2012). VPA induces proliferation of HSC and HPC without inducing apoptosis. Previous studies have demonstrated that expansion of HSC and HPC caused by VPA treatment was accompanied by phenotypic transformation and cellular reprogramming of this primitive cell population (Papa et al., 2018). Besides inducing expression of p53, a tumor suppressor gene that has been shown to promote HSC self-renewal, VPA was also able to protect HSC from damage caused by ROS. Mitochondria, the energy-producing organelles in cells, produce ROS. Elevated levels of ROS can damage DNA and other cellular components, leading to cell death. Interestingly, other antioxidant molecules, such as Levistilide A or Echinochrome A, have also corroborated the impact of controlling ROS levels in HSC and HPC expansion (He et al., 2022; G.-B; Park et al., 2019). Taking advantage of its therapeutic potential, VPA was sponsored in a Phase I clinical trial for hematological malignancies (NCT03885947). Knowing that safety must initially be evaluated using DUCBT, this specific clinical trial divided the cohort of patients into two groups. After demonstrating the safety of the expanded graft in the first group undergoing DUCBT, a second group was subjected to a single-arm HCT with a VPA-expanded graft. Thus, less time was spent transitioning from DUCBT to single-arm UCB HCT.

#### 6.2.2 Pre-clinical molecules

Novel small molecules continue to arise as potential candidates to improve HSC and HPC *ex vivo* expansion, especially after witnessing the near dominance of small molecules in the clinical trial pipeline.

Being enriched in HSC, aldehyde dehydrogenase (ALDH) converts aldehydes into carboxylic acids. This enzyme converts Vitamin A (retinol) to retinoic acid. An early study using 100 μM of diethylaminobenzaldehyde (DEAB), an ALDH inhibitor, showed that differentiation and lineage commitment were blocked in human HSC (CD34^+^CD38^−^Lin^−^) after 7 days of culture, increasing their numbers by around 4-fold (Chute et al., 2005; 2006). The effects of DEAB could be reversed by the co-administration of the retinoic acid receptor (RAR) agonist, all-trans retinoic acid (ATRA), suggesting that the ability of ALDH to produce retinoic acids is essential in determining HSC fate. This study uncovered the modulation of ALDH activity and retinoid signaling as novel and practical strategies to amplify human HSC and HPC. Recognizing the paradoxical challenge of inhibiting ALDH, whose expression in HSC is also a marker of stemness, another expansion study with DEAB led to a more than a 10-fold increase in CD34^+^ALDH^bri^ expression, being coherent with previous studies (Cooper et al., 2018).

Apoptosis contributes to progressive HSC depletion, providing an exciting target for improving the expansion of HSC and HPC by inhibiting apoptotic machinery. Pharmacological inhibition of apoptosis was attempted in UCB-derived CD34^+^ cells expanded with cytokines in the presence or absence of cell-permeable inhibitors of caspases and calpains (i.e., zVADfmk and zLLYfmk, respectively) (Sangeetha, Kale, and Limaye, 2010). Apoptotic protease inhibitors were shown to increase CD34^+^ cell content during *ex vivo* expansion. These cells sustained superior long-term engraftment and efficient regeneration of major lymphoid-myeloid lineages in the bone marrow of non-obese diabetic severe combined immunodeficiency (NOD/SCID) mice compared to cells expanded with growth factors alone.

A recent study uncovered that antagonism of PPAR-γ 3 by GW9662 promoted *ex vivo* expansion of phenotypically and functionally defined subsets of human UCB stem/progenitor cells (Guo et al., 2018). PPAR-γ 3 inhibition was shown to promote HSC and HPC expansion by regulating mitochondrial metabolism and decreasing the capability of HSC differentiation. Functional genomic analysis from this study revealed that HSC expansion and inhibition of differentiation were due to an elevated glycolysis level through the PPARG-FBP1 axis. Eventually, another potent PPARG antagonist, T0070907, and a specific FBP-1 inhibitor, MB05032, were shown to promote the *ex vivo* expansion of UCB-derived progenitors. This shift towards an anaerobic profile leads to better stem cell maintenance. Importantly, one needs to consider the subtle line between whether that shift is due to an attenuation of OXPHOS or an increase in glycolysis (Ivanovic, 2013; Rodriguez et al., 2021).

Bromodomain-containing proteins (BCP) are associated with hematopoietic stemness and HSC self-renewal in mice (Wroblewski et al., 2018; Dey et al., 2019). A recent study screened small molecules targeting various BCP as potential agents for *ex vivo* expansion of human HSC and HPC and identified bromodomain and extra-terminal motif inhibitor CPI203 (Hua et al., 2020). Expanded cells using these small molecules also demonstrated improved engraftment and repopulation in serial transplantation assays. Transcriptomic and functional studies showed that the expansion of long-term repopulating HSC was accompanied by synchronized growth and maturation of megakaryocytes consistent with CPI203-mediated reprogramming of UCB-derived primitive cells.

Kinase inhibitors, typically studied across various cancer pathways, have been investigated regarding their effects on HSC expansion. Glycogen synthase kinase-3 (GSK3) is one of the widely studied kinases involved in many cell functions. GSK3 is responsible for phosphorylating many proteins crucial for HSC fate determination, such as Wnt, Notch, and Hedgehog signaling pathways (Trowbridge et al., 2006). Many significant efforts were made to identify GSK3 inhibitors that can assist in expanding HSC. CHIR99021 was shown to increase hematopoietic recovery in NOD/SCID gamma (NSG) mice, increasing the amount of transplantable HSC 100-fold in combination with insulin by repressing differentiation and expansion (Trowbridge et al., 2006). Two other studies (J. Huang et al., 2012; J; Li et al., 2019) demonstrated that CHIR99201, in combination with mTOR inhibitor rapamycin and p38 inhibitor SB203580, supported the maintenance of functional long-term HSC and promoted HSC expansion, respectively. Rapamycin improved repopulating ability in mice by arresting cell cycle status without affecting apoptosis during expansion (J. Huang et al., 2012). Inhibition of GSK-3b by 6-bromoindirubin-3′-oxime (BIO) upregulated b-catenin in *ex vivo* expanded human CD34^+^ HSC/HPC and promoted early engraftment in NSG mice and engraftment in NOD/SCID mouse models. *In vitro* analysis demonstrated that brief GSK-3b inhibition promoted expansion and provided sustained cell growth and colony-forming activity following withdrawal of the inhibitor. GSK-3b inhibition modulates gene expression in *ex vivo* expanded CD34^+^ enriched cells.

A recent study expanded UCB stem/progenitor cells from non-enriched mononuclear cells (MNC) using novel azole-based small molecules (Bari et al., 2018). The proprietary library of over 50 small molecules was developed using structure-activity-relationship studies of SB203580. A particular analog, C7, resulted in a 1,554-fold increase of absolute viable CD45^+^CD34^+^CD38^–^CD45RA^–^progenitors at least 3.7-fold higher than control cultures. In-depth phenotypic analysis revealed over 600-fold expansion of CD34^+^CD90^+^CD49f^+^ cells and a significant increase of functional colonies from C7-treated cells. Transplantation of C7-expanded UCB grafts into immunodeficient mice resulted in significantly higher engraftment of human CD45^+^ and CD45^+^CD34^+^ cells in PB and BM by day 21 compared to non-expanded and cytokine-expanded grafts.

Prostaglandin E2 has a known role in enhancing HSC expansion. Prostaglandin degrading enzyme, 15-PGDH, reduces the levels of PGE2. Inhibiting 15-PGDH was shown to increase PGE2 levels, helping in improving hematopoiesis (Y. Zhang et al., 2015). Functional genomics and pharmacological inhibition of 15-PGDH demonstrated an increase in the number of HSC and hematopoietic differentiation. An elegant study screening around 230,000 compounds pinpointed a potent 15-PGDH inhibitor (i.e., SW033291) (Y. Zhang et al., 2015). This small molecule increased PGE2 levels and accelerated hematopoietic recovery in mice after bone marrow transplantation. Another compound, SW209415, from the second-generation 15-PGDH inhibitors, also showed increased *in vivo* PGE2 levels and accelerated hematopoietic regeneration after transplantation. SW033291 maintained efficacy even when the transplant donor and recipient were aged, potentiating homing in xenotransplants using human HSC (Desai et al., 2018).

As previously mentioned, the potential of small molecules for the expansion of HSC and HPC is still dependent on the presence of cytokine supplementation. Despite the advantages of small molecule over recombinant cytokines, their range of action typically tends to be more limited, derived from their synthetic origin. Importantly, small molecules may have bioavailability issues that can jeopardize their development. Nicotinamide riboside, which has been mentioned to enhance hematopoietic stem cell function, has poor solubility and limited oral bioavailability (Campbell et al., 2019). While small molecules tend to be more straightforward concerning their signaling impact, as well as manufacturing process, most cytokines have the advantage of having already been extensively studied. This provides cytokines with a body of knowledge that can prove very valuable during development and regulatory approval. A recent study has successfully sidelined cytokine use for stem/progenitor cell expansion, opening the door to cytokine-free systems (Sakurai et al., 2023). Nevertheless, synthetic compounds used in this study lack of long-term safety and efficacy studies in humans. Additionally, they may not be as effective as cytokines in stimulating HSC and HPC expansion, as they may not be able to mimic the complex signaling interactions that occur *in vivo*.

### 6.3 Biomaterial-supported approaches

The incorporation of natural or synthetic biomaterials in devising novel strategies for the expansion of HSC and HPC has been primarily explored (Ingavle, Vaidya, and Kale, 2019). These biomaterials provide structure by granting cell anchorage for possible 3D assembly and control cell organization depending on their distribution or geometry. Biochemical and mechanical cues are also introduced based on the choice of the biomaterial and can be taken advantage of to stimulate cell behavior.

The extracellular matrix (ECM) is an indispensable component of any cell niche, especially in a complex and multilayered environment like the BM. In general, ECM provides structure and plays a significant role in cell signaling, whether through promotion of direct cell-ECM interaction, mechanotransduction or as a harbor for extracellular signaling molecules. Initially conceptualized for whole organ transplantation, isolation of these natural tissue-derived structures from their cellular components was developed and termed decellularization. ECM decellularization for *in vitro* manipulation has been effectively established, evolving from whole organ studies to more simpler cell sheets or even micronized ECM particles for biomaterial incorporation (Brown et al., 2022). Cell type-specific ECM can now be produced and exploited through decellularization for different biomedical applications. Decellularized ECM (dECM) from HS-5 (a human BM stromal cell line) was tested as a substrate for the expansion of HSC and HPC. Native dECM from traditional 2D cultures and spin-coated dECM were directly compared with expansions with a HS-5 feeder layer and without dECM nor HS-5 cells (Wasnik et al., 2016). Spin-coated dECM had a more uniform distribution and doubled the roughness of the native dECM. Interestingly, spin-coated dECM had the best performance, with increased FC in every studied primitive cell population (i.e., TNC, CD34^+^CD45^low^, CD34^+^CD38^−^ and CD34^+^CD133^+^) and CFU colony type. Of note, the quality of produced dECM through traditional *in vitro* culture has been questioned. To boost the benefit of dECM, macromolecular crowding during ECM production has been shown to improve ECM organization, better mimicking *in vivo* conditions (Prewitz et al., 2015). A 7-day expansion of primitive hematopoietic cells on dECM substrates produced with macromolecular crowding consistently showed better results than traditionally made dECM.

Maintaining a biomimetic perspective, hydrogels have provided an attractive avenue to integrate numerous niche factors (e.g., extracellular mechanics, structure three-dimensionality (3D), or matrix turnover) (Madl and Heilshorn, 2018). As water-swollen materials based on polymeric networks, their hydrophilicity, and softer stiffness align with the native environment of HSC and HPC. Hydrogels also have the advantage of being translucid and, therefore, highly compatible with multiple microscopic techniques for observation. Polyethylene glycol (PEG)-based macroporous hydrogels functionalized with adhesive motifs (i.e., Arginylglycylaspartic acid (RGD)) successfully harbored stem/progenitor cells supported by BM MSC. They led to increased expansion compared to traditional two-dimensional (2D) culture surfaces (Raic et al., 2014). In an attempt to take advantage of circulating hematopoietic progenitors, a PuraMatrix™ hydrogel was used as an expansion system (Xu et al., 2022). From a starting population of peripheral blood MNC, an impressive 70 FC in CD34^+^ cells was observed. Notably, while most expansion systems focus on UCB HSC and HPC, this study demonstrated the potential of expanding non-mobilized peripheral blood-derived cells. Still, the most thorough and tailored hydrogel development for HSC expansion was established using a zwitterionic hydrogel (Bai et al., 2019). A super-hydrophilic zwitterionic polymer avoided negative culture artifacts linked to their hydrophobic surfaces, providing an interaction-free scaffold. Polymer links through a polypeptide with metalloproteinase cleavable motifs allowed expanded HSC and HPC to modulate the matrix as desired. After 24 days, encapsulated HSC/HPC maintained their primitiveness (94.6% CD34^+^ expression) and underwent a 322 FC in TNC, preserving long-term repopulating capacity through secondary transplants in mouse models. More recently, a porous hydrogel system was developed for expansion of BM-derived stem/progenitor cells (Liu, Jin, and Wang, 2023). The incorporation of pores was found to reduce ROS production compared to a non-porous control, demonstrating the importance of hydrogel tuning to improve the microenvironment of HSC and HPC during *in vitro* proliferation. Efforts to improve hydrogel culture have also focused on their own matrix stiffness. Acknowledging an age-related increase in stiffness inside the bone marrow, supporting stromal populations (i.e., BM MSC) were seen to reduce their secretion of hematopoietic niche factors. In an attempt to rejuvenate aged murine HSC, BM MSC were cultured together with old HSC in a soft gelatin methacrylate hydrogel (X. Zhang et al., 2023). A recovery of BM MSC production of niche factors was observed with an increase of HSC self-renewal and reversal of HSC aging hallmarks (e.g., myeloid-biased hematopoiesis).

Ultimately, using biomaterials allows the formation of more complex systems for the expansion of HSC and HPC. Constructing hierarchical structures or reaching resemblance with organ-like organization might unlock even more significant expansion potential. An interleaved lattice-mesh structure made of PCL spiked with hydroxyapatite (lattice) and polyurethane (mesh) was coated with vitronectin and seeded with UCB stem/progenitor cells (J. E. Kim et al., 2019). The primitive cell-infused scaffold was then incubated in a vessel capable of applying controlled hydrostatic pressure. Scaffold-adhered HSC and HPC were not indifferent to hydrostatic pressure, outperforming the remaining conditions. Taking advantage of extramedullary niches where HSC naturally go through an extensive expansion phase during development, the fetal liver has been hypothesized to be an exciting alternative to the model. Ferret-derived fetal livers were decellularized and repopulated with human fetal liver-derived stromal cells before infusing UCB HSC and HPC (Mokhtari et al., 2018). This top-down approach harnessed *in vivo* produced ECM with native structure to better recreate the fetal liver niche. 3D fetal liver constructs could better support HSC and HPC expansion than 2D co-cultures, and fetal liver-derived stromal cells outdid other tested repopulation cell types, such as hepatoblasts or BM Stro-1^+^ progenitor stromal cells. To mimic the trabecular bone structure where HSC resides, a porous ceramic scaffold was populated with BM MSC that underwent 3 weeks of osteogenic differentiation (Bourgine et al., 2018). ECM production was promoted, causing the formation of a gel-like film around the ceramic scaffold, renamed engineered niche. After HSC/HPC injection, cells were expanded in a perfusion regimen, reaching 61 FC in TNC. Differentiated compartments were observed inside the engineered niche, similarly to the several microenvironments found in the BM. Future approaches might also take advantage of bone marrow mimicking biomaterials that have been developed for fundamental studies on HSC and HPC and adapt them to expand cells for clinical purposes (Nelson and Roy, 2016; Lipreri et al., 2022).

### 6.4 Co-culture systems

The native environment of HSC and HPC is rich in cell diversity and is responsible for upholding the complex structure of the BM. Different niches exist inside the BM, regulating primitive cell behavior to maintain a healthy steady-state hematopoiesis (Dolgalev and Tikhonova, 2021). Osteoclasts and osteoblasts incorporate the endosteal microenvironment, promoting HSC quiescence. On the other end, co-existing EC lining the marrow vasculature and supportive MSC make up the sinusoidal niche, where HSC are stimulated to increase their proliferative capacity to respond to hematopoietic needs (Méndez-Ferrer et al., 2020). An expected strategy would be recreating the hematopoietic niche *in vitro* by employing cell types that normally populate the BM *in vivo*, especially those involved during hematopoietic regeneration.

Following that rationale, several attempts have been made to expand HSC and HPC using EC. Since primary EC cannot be sustained for many passages *in vitro* without losing their characteristic phenotype or undergoing cell death. E4orf1 protein-derived from adenovirus-36 has been used to stabilize cultured EC (Seandel et al., 2008). EC hematopoietic support was initially shown in murine models to be contact-dependent, and their mechanism was based on AKT-activated expression of an array of endocrine factors, including Notch pro-expansion ligands (i.e., Jagged-1, Jagged-2, DLL1, and DLL4) (Butler et al., 2010). However, the benefit of EC co-culture has been hypothesized to be tissue-dependent (Birbrair and Frenette, 2016). A recently completed clinical trial exploiting this strategy (i.e., NCT03483324—Completed: January 2022) successfully uses human umbilical vein EC (HUVEC) to expand UCB-derived CD34^+^. Interestingly, *E4ORF1* ^*+*^-umbilical arterial EC was shown to promote more significant HSC and HPC expansion, approximately double FC in total nucleated cells (TNC), CD34^+^, CD34^+^CD38^−^ and CD34^+^CD38^−^CD90^+^ cells, compared to co-cultures with HUVEC after 14 days (Li et al., 2020). On the other hand, kidney-derived EC worsened the fold increase of HSC/HPC compared to EC-free conditions (W. Li et al., 2004). Thus, vascular niches have been labeled organ-specific with unique endocrine phenotypes, highlighting the importance of correct EC procurement for hematopoietic stem/progenitor cell expansion by co-culture (Rafii, Butler, and Ding, 2016).

An alternative adherent cell candidate for co-culture would be BM-derived MSC. Their role in promoting HSC and HPC cycling during hematopoietic regeneration has made them a logical target as a co-culture cell type (Mendelson and Frenette, 2014). In fact, MSC have been successfully used as co-adjuvants during HCT, also promoting HSC engraftment (Crippa and Bernardo, 2018). Unlike EC, MSC have significant expansion capacity *in vitro* and have proven to be valuable cell population in cell therapy (Pittenger et al., 2019). MSC manufacturing has seen impressive evolution due to their varied therapeutic properties (e.g., anti-inflammatory, immunomodulatory, and anti-apoptotic). Numerous scalable expansion platforms have been developed and required reagents have been improved to uphold ever more stringent regulatory standards (e.g., xeno-free formulations or chemically-defined media) (Olsen et al., 2018; Pigeau, Csaszar, and Dulgar-Tulloch, 2018; Almeida et al., 2019). As with EC, MSC can be sourced from various tissues, with most having been studied for their population-specific hematopoietic support capacity (Klein et al., 2013; Bucar et al., 2021). Importantly, the use of MSC as an allogeneic source for hematopoietic cell co-culture expansion was demonstrated early on (Gonçalves et al., 2006). Interactions between HSC/HPC and MSC were also seen to be contact-dependent (Jing et al., 2010; Silva et al., 2010; Walenda et al., 2011). These same interactions were shown to delay concomitant differentiation of HSC and HPC during expansion compared to liquid cultures using only exogenous cytokines (Branco et al., 2020). Notably, maintenance of SCID repopulating cells (SRC) was demonstrated when including an MSC feeder layer during expansion of HSC and HPC (Hammoud et al., 2012). Still, expansion dynamics during this type of co-culture are complex. After sedimentation, HSC/HPC that maintain contact with supportive MSC become the main drivers of proliferation (Jing et al., 2010). A more differentiated non-adherent population forms as progeny of adherent HSC and HPC through cell egress (Jing et al., 2010). A fraction of adherent HSC and HPC eventually migrates beneath the MSC feeder layer, proliferate slower, and become more enriched in CD34^+^CD38^−^ cells (Jing et al., 2010). Interestingly, reported HSC and HPC behavior is comparable to the native BM, with the formation of spatially and functionally distinct cell populations (i.e., more primitive cells under MSC versus proliferative cells over MSC; quiescent HSC located in the endosteal niche versus proliferative HSC/HPC in the sinusoidal niche). By creating such a biomimetic effect with a single supportive cell type, HSC and HPC expansion through MSC co-culture has originated two clinical trials, starting with unselected UCB MNC (Lima et al., 2012). An initial trial with 31 patients suffering from hematological cancers studied the impact of using a pre-expanded unit through co-culture with BM MSC during DUCBT (Lima et al., 2012). A median fold change of 30.1 was obtained for CD34^+^ cells, contributing to an impressive neutrophil engraftment of 15 days compared to 24 days registered in unmanipulated DUCBT. A second clinical trial attempted to combine cord blood unit expansion using the co-culture strategy in addition to fucosylation, a homing-improvement strategy (NCT03096782 - Completed). After treating a cohort of 6 patients, recent results note a median neutrophil engraftment of 25.5 (NCT03096782, n.d.). Although having reached the clinical trial pipeline, the inclusion of supporting feeder cells always adds a layer of complexity that might delay obtaining regulatory approval. Some have gone further and attempted MSC augmentation or revitalization, building on the natural advantage of expanding HSC and HPC on MSC feeder layers *in vitro*. BM-derived MSC were transduced with lentiviral vectors expressing angiopoietin-like-5 (previously shown to also promote HSC and HPC expansion when used exogenously in culture (C. C. Zhang et al., 2008)), leading to a 60-fold increase in SRC (Khoury et al., 2011). Another strategy focused on revitalizing BM-derived MSC that typically lose their niche-promoting properties after *in vitro* adaptation (Nakahara et al., 2019). Five transcription factors were identified in an RNA sequencing screen that were able to recover expression of their niche-promoting factors, resulting in around a 4-fold increase in HSC numbers compared to non-revitalized MSC (Nakahara et al., 2019).

### 6.5 Ligand immobilization

Different methods have been used to tap into signaling pathways that drive the expansion of HSC and HPC. Although the use of ligands was early explored and simple to incorporate, some were observed to require structural stability to enhance their effect (A. Peled et al., 1999; Varnum-Finney et al., 2000). The Notch-mediated expansion system was developed through ligand immobilization. Specifically, Notch ligand delta-like ligand 1 (DLL1) was fused to the Fc portion of human IgG-1 (Delta1^ext−IgG^) and immobilized onto plastic culture ware, being able to support stem/progenitor cell expansion (Delaney et al., 2005). Initial efforts in translating this system led to the development of a clinical trial to evaluate the applicability of expanded HSC and HPC in DUCBT (NCT00343798) (Delaney et al., 2010). Although rapid myeloid reconstitution was observed, persistent long-term engraftment was mainly assured by the unexpanded graft. In fact, after 16 days of culture, the expression of CD34 was down to 14.5%, indicating the presence of more differentiated populations (Delaney et al., 2010). This result was expected since the Notch signaling pathway is also highly associated with T cell development and involved in regulating B cell differentiation (Ng et al., 2021; Vanderbeck and Maillard, 2021). Acknowledging the limitations of this particular system in what concerns long-term engraftment and reconstitution, a shift in therapeutic purpose was introduced. Instead of pursuing long-term reconstitution, requiring more primitive HSC, expanded stem/progenitor cells deriving from this expansion system could be employed to facilitate/accelerate immune reconstitution after an HCT, a major post-transplant constraint. Multiple clinical trials have explored the applicability of such a therapeutic approach (Table 1), sponsored initially by a Seattle-based spin-off, Nohla Therapeutics. In the meantime, as a technological platform, this expansion system has been transferred to the proprietary portfolio of Deverra Therapeutics. Nevertheless, attempts were made to maintain undifferentiated populations by combining Delta1^ext−IgG^ coatings with hypoxia, limiting endoplasmic reticulum stress (Araki et al., 2021). By adding hypoxia (1%–2% O_2_), long-term HSC experienced a 4.2 FC compared to the same experimental system in normoxia (Araki et al., 2021).

### 6.6 Bioreactors

For cell and gene therapies, translation to bioreactor systems is deeply encouraged, since their scalability allows reaching clinically-relevant cell numbers coupled with processing control and automation (Lipsitz, Timmins, and Zandstra, 2016). These vessels facilitate reliable manufacturing since they take advantage of feedback from sophisticated monitoring instruments to maintain production homeostasis. Early application of bioreactor systems in hematopoietic cell expansion saw the arrangement of two clinical trials, one using BM MNC for autologous transplants and another evaluating expansion of UCB MNC for HCT (P. Stiff et al., 2000; Jaroscak et al., 2003). Hematopoietic stem/progenitor cells were expanded in an AastromReplicell System, where stromal populations (only present in BM MNC) can form an adhesive supportive layer *in situ*. A feeding regimen based on perfusion allows a continuous exchange of nutrients and metabolites while maintaining cells inside. No advantage in reconstitution timeframes was observed, although these trials were pivotal in demonstrating the safety of expanded cells (Jaroscak et al., 2003).

The benefit of active monitoring of secreted factors negatively affecting the expansion of HSC and HPC has been demonstrated. Through computational modeling, a fed-batch routine was hypothesized to increase cell expansion levels due to the dilution of paracrine factors secreted by differentiating cells that accumulate over time (Csaszar et al., 2012). These factors (e.g., TGF-β1, CCL2 or CXCL12) were seen to be inhibitory towards self-renewal of more primitive HSC. Between several media manipulation techniques (i.e., medium exchange at different intervals, perfusion or fed-batch), fed-batch was experimentally validated as the best mode of operation. Real-time monitoring of individual secreted factors through quantum dot labeling allowed for continuous adjustment of dilution rates, improving primitive cell proliferation even more (Csaszar et al., 2014). Further development of a proportional-integral-derivative (PID) controller optimized the feedback control system, showcasing the impact monitoring can have on HSC and HPC expansion (Caldwell, Wang, and Zandstra, 2015).

Recently, a hollow-fiber perfused bioreactor (Quantum, by Terumo BCT) was used as a platform for HSC and HPC expansion (Jones et al., 2022). As an automated platform, labor times are significantly reduced, and medium changes are performed through a perfusion system. A central chamber lined with a membrane separates it into two cavities (hollow fiber membrane module). The dividing membrane was coated with fibronectin and SDF-1, adding an ECM element and simulating chemotactic homing in the system to support the stem/progenitor cell increase in the bioreactor lumen. An 8-day expansion from a starting inoculum of 2 × 10^6^ CD34^+^ cells yielded 51 fold increase in the same population, largely overcoming minimal doses for single cord and DUCBT (Dehn et al., 2019). Existing differentiation was predominantly towards the myeloid lineage, namely, immature neutrophils and platelets.

Of note, upgrading existing expansion strategies by adapting them into a bioreactor setting should be naturally aspired. An abovementioned biomaterial-based system, where a ceramic scaffold was populated with BM MSC that were differentiated into the osteogenic lineage and repopulated with HSC and HPC (Bourgine et al., 2018), has recently been translated to a customizable 3D printed perfusion bioreactor (Dupard, Garcia, and Bourgine, 2023). After scaffold colonization with a MSC cell line stably expressing hTERT-iCasp9 (i.e., Mesenchymal Sword of Damocles (MSOD)), 1 week culture after seeding of CD34^+^ cells led to a 98 FC in CD90^−^EPCR^+^ multipotent progenitors and a 13 FC in CD34^+^CD45RA^−^CD90^+^EPCR^+^ HSC. As expected, some stimuli have not yet been sufficiently explored and incorporated in bioreactors for HSC and HPC expansion. In the bone marrow, cells are subjected to several mechanical stresses, namely, shear stress, circumferential strain as well as hydrostatic pressure (Li et al., 2021). These biomechanical cues have been identified as critical regulators of the hematopoietic homeostasis. Bioreactor systems featuring these mechanical cues should be developed for the expansion of stem/progenitor cells. When possible, existing bioreactor configurations that perform mechanical loading, such as the versatile tissue growth and remodeling (Vertigro) bioreactor (Kelle et al., 2017), should be adapted.

## 7 State-of-the-art on potency assays for HSC and HPC

With cell and gene therapy products based on expanded HSC and HPC on the cusp of regulatory approval and market authorization, roadmaps for a thorough evaluation of stem/progenitor cell potency are still lacking. The scarcity of means to measure therapeutic action spreads throughout most cell and gene therapies, contrasting with traditional pharmaceuticals and biopharmaceuticals (Kirouac and Zandstra, 2008). The inherent complexity of living cells as a medicinal product has made the development of functional assays difficult. The therapeutic MoA, which serves as the footing for potency measurements (a vital quality attribute), is often partially unknown (Bravery et al., 2013). In cases where *in vitro* or *in vivo* functional assays are available, they may not be adequate for quality control of cell-based manufacturing. Extensive read-out waiting periods, fragile reproducibility, or high dependence on operator capability are some of the concerns that affect these critical quality and security checkpoints (Bravery et al., 2013).

Decades of HCT with minimally manipulated HSC/HPC have relied on cell dose (TNC), cell viability, CD34 expression, and HLA-matching as selection criteria (Rich, 2015). None of these factors are quantitative measures of biological function, as stated the definition of cell potency (Lipsitz, Timmins, and Zandstra, 2016). The abovementioned features are, in fact, simply correlative with the therapeutic effect of stem/progenitor cells. To tackle this issue, a recent survey by the Association for the Advancement of Blood & Biotherapies (AABB) listed existing potency testing for cryopreserved HSC and HPC (Reich-Slotky et al., 2022). Viable CD34^+^ cells (53%), expression of aldehyde dehydrogenase (ALDH) (5%), CFU (56%), and patient engraftment (5%) were the only assays to be mentioned. The CFU assay is the most representative assessment of hematopoietic repopulation potential included in this survey. With the development of standardized and commercial semi-solid methylcellulose-based medium (i.e., MethoCult™), the CFU functional assay for quantification of HPC myeloid differentiation potential was developed. By quantifying different myeloid and erythroid colonies (CFU-granulocyte, erythroid, macrophage, megakaryocyte (CFU-GEMM), burst-forming unit-erythroid (BFU-E) and CFU-granulocyte-macrophage (CFU-GM)) after 2 weeks, total CFU FC indicates progenitor enrichment. In contrast, the ratio between different colony types may provide signs of HPC lineage priming (Pamphilon et al., 2013). However, its 2-week duration combined with significant subjectivity and inter-operator variability associated with manual colony classification, renders it less than ideal for potency testing (Pamphilon et al., 2013). Advances in automatic quantification equipment (i.e., STEMvision™) or the development of faster and more reliable flow-based alternatives (i.e., quantification of ALDH expression or IL-3 mediated STAT5 phosphorylation) have tried to address these drawbacks (Shoulars et al., 2016; Simard et al., 2019; VELIER et al., 2019). Intriguingly, ALDH expression explored as a surrogate for CFU did correlate well with CFU readouts and the engraftment of a small cohort of 78 patients (Shoulars et al., 2016). If adopted as a potency assay, ALDH expression must be co-expressed with HSC characteristic immunophenotype (e.g., CD34^+^CD45RA^−^CD90^+^) to assure specificity and does not relay differentiation potential readouts. Some have proposed telomere length as a potency assay for HSC and HPC. Similar to other stem cell compartments, HSC have DNA elongation mechanisms in place to avoid genomic damage throughout the lifespan of the organism (Blasco, 2007), drawing a distinction from more differentiated populations (Hills et al., 2009). As such, measurement of telomere length has been used as a readout of *ex vivo* HSC expansion, namely, to ascertain the genomic quality of expanded HSC and to rule out unwanted differentiation (Yao et al., 2006; Andrade et al., 2010; Wagner et al., 2016). However, if trying to ascertain replicative stress on long-term HSC, their very low proportion within the whole expanded cell population, comprising also committed progenitors and more mature cells, may hinder the quantification of their telomere length. Recently, a co-culture assay was developed to assess HSC self-renewal *in vitro* in a biomimetic niche. Taking advantage of the improved microenvironment when including an MSC feeder layer, primitive cell fitness is determined comparing a co-culture expansion with a control condition where cells are expanded without an MSC feeder layer (Branco et al., 2022). Lastly, long-term engraftment constitutes the ideal measurement of HSC potency for transplantation, yet a potency assay must provide readouts before administration to evaluate product quality before treatment. Although a surrogate assay exists involving transplantation of human HSC into a NOD/SCID or NSG mouse model, it also suffers from extensive waiting periods (4–6 weeks for primary transplantation and 10–12 weeks for secondary transplants) (C. Y. Park, Majeti, and Weissman, 2008). On the other hand, it may be limited to the detection of short-term reconstitution (M. Ito et al., 2002).

Overall, the lack of robust potency assays that meet translation requirements is aggravated by changes in premises that are applicable for minimally manipulated stem/progenitor cells, but not for expanded cells. For example, CD38, as a surface marker co-expressed with CD34, has long been used as a surrogate to characterize hematopoietic stemness (i.e., CD34^+^CD38^−^ - more primitive; CD34^+^CD38^+^ - more differentiated). In expanded stem/progenitor cell populations, a dissociation between the expression of this surface marker and its expected phenotype was observed, alerting to assumptions made based on knowledge of freshly isolated HSC and HPC or *in vivo* characterizations (Dorrell et al., 2000). Adding to this issue, expansion-validated surface markers might also be expansion system-specific, depending on which pathway is triggered. UM171-expanded cells was seen to upregulate the expression of EPCR (Fares et al., 2014). EPCR^+^ cells were shown to have extensive self-renewal capacity and gather most of the repopulation potential when transplanted into mouse models, sitting at the top of the tiered organization of *ex vivo* expanded HSC (Fares et al., 2017). Although it has been defined as a reliable and robust marker for expanded HSC, its expression is not transversal to every expansion strategy, evidencing the existence of strategy-specific markers (Fares et al., 2017; Papa et al., 2020).

These limitations, which are in part shared between cell and gene therapies while others are specific for expanded HSC and HPC, must be confronted with novel methodologies. An OMICS approach provides a global and systematic insight into cell machinery. Multi-dimensionality would offer more robustness since dependence on a handful of discrete biomarkers is vulnerable to culture-induced changes (e.g., CD38 expression). Even if the MoA is not fully described, a comparison between expanded cells and their ideal freshly isolated counterparts would still be possible, allowing potency determination and product quality assurance. Also, if wanting to refine from a broader therapeutic effect derived from HCT, more restricted gene sets linked to BM engraftment, myeloid reconstitution, or T cell compartment recovery may be investigated at a transcriptomic level. Bioinformatic algorithms have been developed to estimate the differentiation potential from transcriptomes, potentially substituting CFU readouts (Teschendorff and Enver, 2017). Promising expectations were verified when differences in manufacturing two clinically relevant regulatory macrophage populations were uncovered by transcriptomics after traditional characterizations, namely, surface marker expression and cytokine production, could not distinguish both products (Gurvich et al., 2020). This far-reaching cellular scrutiny can also be valuable at the protein level. Proteomic profiling of MSC from different sources was performed to ascertain the most angiogenic population, allowing for a more comprehensive evaluation of MSC potency (Kehl et al., 2019). For expanded HSC and HPC, secretome analysis might be able to show signs of lineage priming, predicting clinical outcomes.

Potency characterization for expanded stem/progenitor cells is a relevant challenge in their translation. Few potency assays exist that can qualify for manufactured product quality control. The therapeutic benefit in HCT is multifactorial and complex. It involves migration to the BM, HSC engraftment in their rightful niche, timely long-term hematopoietic reconstitution (i.e., steady-state hematopoiesis), and stabilization of the HSC pool. Consequently, functional assay development is hampered, exacerbated by hard-to-mimic biological functions that are difficult to translate to an *in vitro* assay. Assay development should opt for a global approach or a breakdown of individual therapeutic sections. Significantly, expanded HSC and HPC have been exposed to *in vitro* culture, and care should be taken when extrapolating knowledge (including biomarkers) from *in vivo* or freshly isolated cells.

## 8 Conclusion

HCT holds significant promise as a potential cure for various blood disorders, contingent upon the availability of an ample supply of primitive cells from compatible donors. Recognizing the challenges associated with the scarcity and variability of HSC, a myriad of strategies have been devised to enhance their *ex vivo* expansion. These strategies encompass diverse approaches such as utilizing alternative sources of HSC and HPC, including mPB, manipulating the microenvironment to mitigate GVHD, and advancing our comprehension of HSC biology and regulation.

Recent strides in HSC research have unveiled the intricate roles played by signaling pathways mediated by cytokines and growth factors, cell cycle regulation, transcription factors, and epigenetic modifiers, as well as the metabolic regulation of HSC in self-renewal and differentiation. In addition, the intricate interplay between HSC and the aging process encompasses a variety of changes in intrinsic properties, niche dynamics, inflammation, clonal hematopoiesis, metabolism, and underlying mechanisms. This understanding is pivotal for devising strategies to harness HSC potential in regenerative therapies and mitigate age-associated hematopoietic deficiencies.

Notably, EV have emerged as an innovative tool for the expansion of primitive hematopoietic cells, leveraging their ability to transfer bioactive molecules and modulate HSC fate. Furthermore, the incorporation of clinically relevant small molecules, biomimetic substrates, and co-culture systems has enabled the fine-tuning of HSC therapy for its meticulous pre-clinical evaluation.

In tandem with these advancements, bioreactor systems have been purposefully designed to facilitate the large-scale expansion of HSC and HPC while preserving their quality and functionality. These developments collectively open up new avenues for enhancing the outcomes and accessibility of HCT for patients grappling with hematological diseases.

Nevertheless, the landscape of hematopoietic stem/progenitor cell therapy is not without its challenges. Foremost among them is the challenging task of securing a compatible donor for patients, particularly those afflicted with rare or complex diseases. Additionally, there is the ongoing challenge of navigating the complexities and risks inherent in the transplant procedure, encompassing issues like GVHD, infections, graft failure, infertility, organ damage, and mortality. These adverse events not only diminish the survival rates but also impact the overall quality of life for patients, necessitating vigilant and intensive care.

Moreover, genome editing of HSC and HPC has been developed in the context of several conditions of the hematopoietic system with important advances as well as ongoing challenges in the field (reviewed in (Bhoopalan et al., 2023)). Harnessing these technologies is dependent on the feasibility of scaling-up the manufacturing and delivery of stem/progenitor cells, including those genetically modified *ex vivo*, to meet the burgeoning clinical demand. This implies the establishment of standardized and optimized protocols, stringent quality control measures, regulatory approvals, and robust logistical support to ensure the consistent availability and accessibility of stem cell products.

Closely associated with the manufacture of expanded HSC and HPC, regulatory requirements and their rapid evolution are also a risk for the viability of a potential stem/progenitor-based product. Currently, compliance with established Current Good Tissue Practices (cGTP) guidelines is needed for the use of autologous cells. Conversely, allogeneic cells, derived from a distinct donor, demand compliance with more stringent cGMP standards. While a degree of overlap exists between certain aspects of cGTP and cGMP, a worrying trend has been identified (Cundell, Atkins, and Lau, 2023). This trend suggests a deficiency in the implementation of essential aseptic processing controls and robust environmental monitoring programs within routine clinical practice. This inconsistency may be attributed, at least partially, to the insufficient level of detail provided within established professional standards. Additionally, a potential lack of rigorous oversight by regulatory and accreditation bodies may be a contributing factor.

By providing a novel option of treatment for HCT patients, namely, the infusion of previously expanded HSC and HPC, ethical concerns may arise. Specifically, the current informed consent process for HCT will need a critical reevaluation. The inherent uncertainties surrounding this complex treatment demand a multifaceted approach that caters to both individual and general understanding of risks and various medical considerations. A recent review identifies three key areas where the process currently falls short. These include: a) Sociocultural Competence: The system may not adequately address the unique needs and communication styles of patients from diverse backgrounds; b) Financial Burden Preparedness: Patients may be ill-prepared for the substantial financial impact of HCT; and c) Patient and Caregiver Preparedness: Both patients and caregivers may enter the transplantation process without a clear understanding of what to expect (Cusatis et al., 2023). These shortcomings can be traced back to an inadequately designed informed consent process. This review proposes solutions that converge on a common theme: a shift towards a more patient-centered informed consent process. The core ethical principles of patient autonomy and institutional/physician legal protection underpinning informed consent remain vital. However, the way this process is executed needs to evolve.

Moreover, concerning market access challenges of advanced cell therapies, their cost-effectiveness would largely benefit from decision-support tools through the assessment of both economical and operational aspects of cell manufacturing (Sarkis et al., 2021).

Addressing these challenges is crucial for unlocking the full potential of hematopoietic stem/progenitor cell therapy and realizing its transformative impact on the treatment of blood disorders.
